# Ancient genomes reveal early-stage admixture and genetic diversity in the Northwestern Kyushu Yayoi

**DOI:** 10.1038/s41598-026-34996-7

**Published:** 2026-01-07

**Authors:** Jonghyun Kim, Fuzuki Mizuno, Takayuki Matsushita, Masami Matsushita, Izumi Naka, Kunihiko Kurosaki, Fuyuki Tokanai, Shintaroh Ueda, Jun Ohashi

**Affiliations:** 1 Department of Biological Sciences, Graduate School of Science, The University of Tokyo, 7-3-1, Hongo, Bunkyo-ku, Tokyo, 113-0033 Japan; 2 Department of Legal Medicine, Toho University School of Medicine, 5-21-16, Omori-Nishi, Ota-ku, Tokyo, 143-8540 Japan; 3 The Doigahama Site Anthropological Museum, Yamaguchi, 759-6121 Japan; 4 Center for Accelerator Mass Spectrometry, Yamagata University, Kaminoyama, 999-3101 Japan

**Keywords:** Admixture, Ancient DNA, Japanese ancestry, Northwestern kyushu yayoi, Evolution, Genetics

## Abstract

The demographic history of the Japanese archipelago was shaped by major episodes of migration and admixture, most notably the transition approximately 3,000 years ago from the Jomon period, a long-established hunter-gatherer tradition, to the Yayoi period, when migrants from the Eurasian continent introduced agriculture and new technologies. However, the timing, extent, and regional variability of the admixture between continental migrants and indigenous Jomon populations remain poorly understood. Northwestern Kyushu has drawn particular attention because skeletal analyses have indicated that Yayoi individuals from this region retain Jomon-like morphological features. However, their genetic background remains unclear. Here, we report whole genome sequences from four Northwestern Kyushu Yayoi individuals excavated from Nagasaki Prefecture, Japan. The two individuals retained nearly full Jomon ancestry, demonstrating that unadmixed Jomon descendants persisted alongside the admixed populations until the transitional phase between the Early and Middle Yayoi periods. The other two individuals showed clear evidence of an admixture, and genetic analyses indicated that gene flow between migrants and the indigenous Jomon population had already begun in this region approximately 2.5–2.6 kya. These findings suggest that the admixture in northwestern Kyushu occurred gradually rather than through a single large-scale migration event at the onset of the Yayoi period, offering new insights into the complex demographic dynamics that shaped the genetic landscape of ancient Japan.

**Supplementary Information:**

The online version contains supplementary material available at 10.1038/s41598-026-34996-7.

## Introduction

Understanding how distinct human populations admix and form new genetic and cultural identities is a central question in population history. Episodes of migration and admixture have repeatedly shaped human diversity worldwide, influencing genetic ancestry as well as social, cultural, and technological transformations. For example, population movements accompanying the spread of agriculture during the Holocene fundamentally reshaped the genetic landscapes of regions such as Europe, South Asia, Southeast Asia, and Africa. The Japanese archipelago offers a particularly well-documented case study of such a transformation, in which a long-lasting hunter-gatherer culture was replaced by an agricultural society through migration and admixture.

The prehistory of the Japanese archipelago is divided into two major cultural periods: Jomon and Yayoi. The Jomon period (ca. 16,500 years ago to ca. 10th century BCE) represents a Neolithic society characterized by foraging, fishing, and the production of distinctive “cord-marked” pottery^1^. Archaeological and genetic evidence indicate that the Jomon people maintained a unique cultural and biological identity for millennia, largely isolated from the Eurasian continent^1–4^. In contrast, the Yayoi period, beginning around the 10th century BCE and named after the site where its characteristic pottery was first identified, marks the introduction of wet rice agriculture and new technologies by migrants from continental East Asia, most likely via the Korean Peninsula^5–7^.

This migration led to admixture between the indigenous Jomon and incoming continental populations, resulting in major demographic, cultural, and technological transformations. This admixture is widely regarded as the primary process shaping the ancestry of modern mainland Japanese, often summarized in the literature as the “Dual Structure Model”^8^, and population genetic studies have firmly established that the continental ancestry component in modern Japanese and Yayoi individuals derives mainly from groups related to the Korean Peninsula^9–12^.

These findings raise the questions of where and how the initial arrival of migrants from the Korean Peninsula occurred within the Japanese archipelago. Archaeological evidence indicates that northern Kyushu was the earliest region to adopt wet rice agriculture^5^ and exhibits numerous Korean-style artifacts^6^. Because the Korean Peninsula and northern Kyushu are separated by only a narrow strait, this region, including Nagasaki Prefecture, is widely regarded as the primary entry point for continental migrants. In particular, the Tsushima and Iki Islands, located between the peninsula and mainland Kyushu, are considered stepping stones along this migration route^7^. However, although northern Kyushu as a whole served as the major gateway for these migrants, archaeological and morphological evidence suggest that the demographic impact of continental ancestry was not uniform across the region, with northwestern Kyushu exhibiting particularly distinctive patterns.

The northwestern region of northern Kyushu has attracted considerable attention owing to the distinctive morphological traits of the remains found in this region. Analyses of ancient human skeletal remains have distinguished the people of the Yayoi period in northwestern Kyushu (referred to here as the “Northwestern Kyushu Yayoi”) from those in other regions. Northwestern Kyushu Yayoi exhibit morphological features resembling those of the Jomon people, such as a low, wide face and short stature^13–15^. In contrast, Yayoi individuals from surrounding regions, such as the Northern Kyushu Yayoi and those from the Doigahama site in Yamaguchi Prefecture, located across the Kanmon Strait from Kyushu, display greater stature and increased facial height^16,17^. Although these morphological characteristics are well-documented, the genetic background of Northwestern Kyushu Yayoi remains poorly understood.

A recent ancient DNA study provided important initial insights by analyzing two Northwestern Kyushu Yayoi individuals from the Shimomotoyama rock shelter site, revealing substantial genomic affinity between the Northwestern Kyushu Yayoi and the Jomon, despite the presence of continental Asian ancestry^18^. However, these conclusions are derived from only two individuals from a single locality, even though numerous Yayoi archaeological sites are known in this region. To achieve a more comprehensive understanding of the genetic diversity and population structure of the Northwestern Kyushu Yayoi, genomic data from a broader range of sites and individuals are required.

**Fig. 1:**
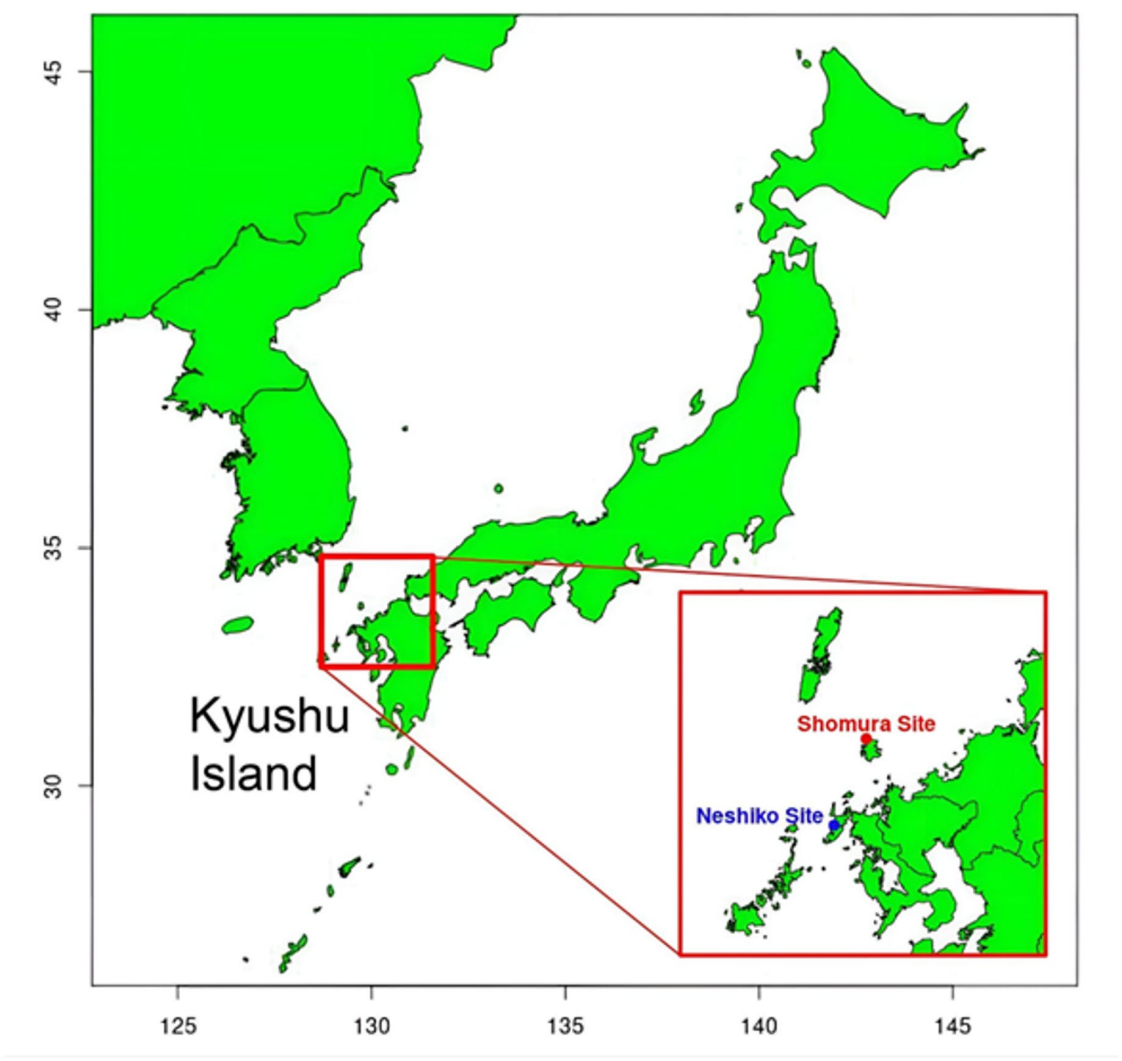
Geographical location of the excavation sites for the newly analyzed samples. Location of Kyushu Island within the Japanese archipelago. Neshiko and Shomura, the excavation sites of the analyzed Northwestern Kyushu Yayoi individuals, are highlighted by red rectangles.

To address this issue, we sequenced Yayoi genomes from two additional archaeological sites associated with Northwestern Kyushu Yayoi: one from the Shomura site on Iki Island^19^ and three from the Neshiko site on Hirado Island^20,21^ (Fig. 1). The individuals excavated from Neshiko exhibited morphological features similar to those of Jomon, consistent with previous reports, whereas the individuals from Shomura could not be definitively classified due to incomplete skeletal remains (Figure S1). Through comprehensive population genetic analyses, we characterized the genetic profiles of these Northwestern Kyushu Yayoi individuals, quantifying the extent of their retained Jomon ancestry and estimating the timing of admixture based on individuals who carry ancestry derived from migrants.

## Results

### Chronological and dietary context of the Yayoi individuals

To establish the temporal and dietary contexts of Yayoi individuals, we performed radiocarbon dating and stable isotope analyses of bone collagen to clarify when they lived and the subsistence strategies they practiced during the early Yayoi period. The measured atomic carbon and nitrogen ratios (C/N), δ^13^C and δ^15^N values, ^14^C ages, and calibrated ages of the four bone collagen samples are summarized in Table S1. The C/N ratio range from 3.3 to 3.7. Three of these values were within the recommended range (2.9–3.6) for well-preserved bone collagen^22^, suggesting that the extracted collagen samples were suitable for radiocarbon dating. The value for sample MSMR-No5 (Shomura) deviates slightly from the recommended range, possibly indicating minor contamination by biogenic substances such as humic acids. The δ^13^C values range from − 16.3‰ to − 13.5‰, with a mean of − 15.1‰ and a standard deviation (SD) of 1.2‰. The δ^15^N values range from 10.6‰ to 16.7‰, with a mean of 13.2‰ and an SD of 2.7‰. Figure S2 shows the relationship between δ^13^C and δ^15^N isotope values in bone samples, compared with reference data reported by Yoneda et al.^23^. These values are higher than those expected for terrestrial herbivore consumption. This suggests that individuals may have relied not only on terrestrial ecosystems, but also on protein sources with higher nitrogen isotope ratios, such as aquatic resources or rice-based agricultural products.

The radiocarbon (^14^C) ages of the four samples ranged from 2,441 ± 20 year before present (BP) to 2,227 ± 20 year BP (Table S1). Since these humans obtained part of their dietary proteins from marine resources, bone collagen contains a proportion of marine-derived carbon. Therefore, the marine reservoir effect should be considered when obtaining accurate calendar ages from collagen-based radiocarbon dates. The proportion of marine resources in each collagen sample was estimated from the carbon isotope ratio (δ¹³C), following the methodology of Arneborg et al.^24^. These estimated values were used to construct a mixed calibration curve based on the IntCal20^25^ and Marine20^26^ datasets, and calibration was performed using OxCal. The local reservoir effect was corrected using a ΔR value of − 296 ± 35^27^, as employed in the analysis of human bones from the Otomo site in the same Saga Prefecture^28^. Figure S3 shows the probability distributions of the calibrated dates for the four collagen samples. The 95.4% probability ranges of the calibrated calendar dates are as follows: NSK–11 (Neshiko 11), 345–281 cal BC (7.8%) and 258–34 cal BC (87.6%); NSK–13 (Neshiko 13), 392–149 cal BC; NSK–16 (Neshiko 16), 396–166 cal BC; and MSMR-No5 (Shomura), 506–184 cal BC. The median calibrated ages of the samples ranged from 348 to 148 cal BC.

### Maternal and paternal lineages reflecting dual origins

To assess the maternal and paternal genetic lineages of the Yayoi individuals and evaluate potential contributions from both continental migrants and indigenous populations, we analyzed the mitochondrial DNA (mtDNA) and Y-chromosome haplogroups of the four Yayoi individuals (Table 1). The mtDNA haplogroup of the Shomura Yayoi sample from Iki Island was identified as D4c1a, whereas the Y chromosome belonged to the O1b2a1a1a lineage. Both mtDNA haplogroup D4 and Y chromosome haplogroup O1b2 (formerly O2b) are common lineages not only among the Japanese, but also among continental East Asians, including Koreans, Chinese, and Mongolians^29,30^. O1b2a1a1 (formerly O2b1) is prevalent in Japan and Korea^30^. These haplogroups may have been introduced into the Japanese archipelago from continental East Asia during the Yayoi period. Because the mtDNA haplogroups typically observed in Jomon individuals are M7a and N9b^31,32^, the presence of haplogroup D4c1a in the Shomura individual suggests that this person carried continental ancestry and was likely a descendant of admixed populations of migrants and indigenous Jomon people.

**Table 1:** Estimated MtDNA and Y chromosome haplogroups of Northwestern Kyusyu Yayoi samples.

	Genetic sex	mtDNA	Y chromosome
Shomura	Male	D4c1a	O1b2a1a1a
Neshiko 11	Male	M7a1a	D1a2a1c1
Neshiko 13	Male	M7a1a	D1a2a1c1
Neshiko 16	Female	M7a1a	-

In contrast, the mtDNA of three Northwestern Kyushu Yayoi individuals from the Neshiko site was identified as belonging to haplogroup M7a1a, whereas two male individuals carried the Y chromosome haplogroup D1a2a1c1. Both mtDNA and Y chromosome haplogroups are rare in continental Eurasia but are frequently found in present-day Japanese populations and are considered to be associated with Jomon ancestry haplogroups^31,33–35^. It is worth noting, however, that one of these individuals (Neshiko 11) carries continental-derived ancestry according to our nuclear genome analyses, highlighting that uniparental markers do not necessarily capture the full complexity of admixture history.

### Kinship relationships among individuals

To establish whether they represented independent members of the population or closely related family members, we calculated the kinship coefficients (KING-robust^36^ among the four Yayoi individuals (Table 2). Neshiko 13 and Neshiko 16 may have a third-degree familial relationship, because their kinship coefficient (0.0801138) falls between 0.08839 and 0.04419. This third-degree relationship was further supported by an IBD1 value of 0.264 for this pair, whereas all other pairs exhibited extremely low IBD1 and IBD2 values (Table S7). Because all six pairs of the four Yayoi individuals exhibited kinship coefficients below 0.08839, confirming the absence of second-degree familial relationships, we included all four Yayoi individuals in subsequent population genetic analyses.

**Table 2:** KING kinship coefficient.

	Shomura	Neshiko 11	Neshiko 13	Neshiko 16
Shomura				
Neshiko 11	−0.00798235			
Neshiko 13	−0.102258	−0.0850009		
Neshiko 16	−0.116183	−0.0941535	0.0801138	

### Genetic affinities of Northwestern Kyushu Yayoi individuals

**Fig. 2:**
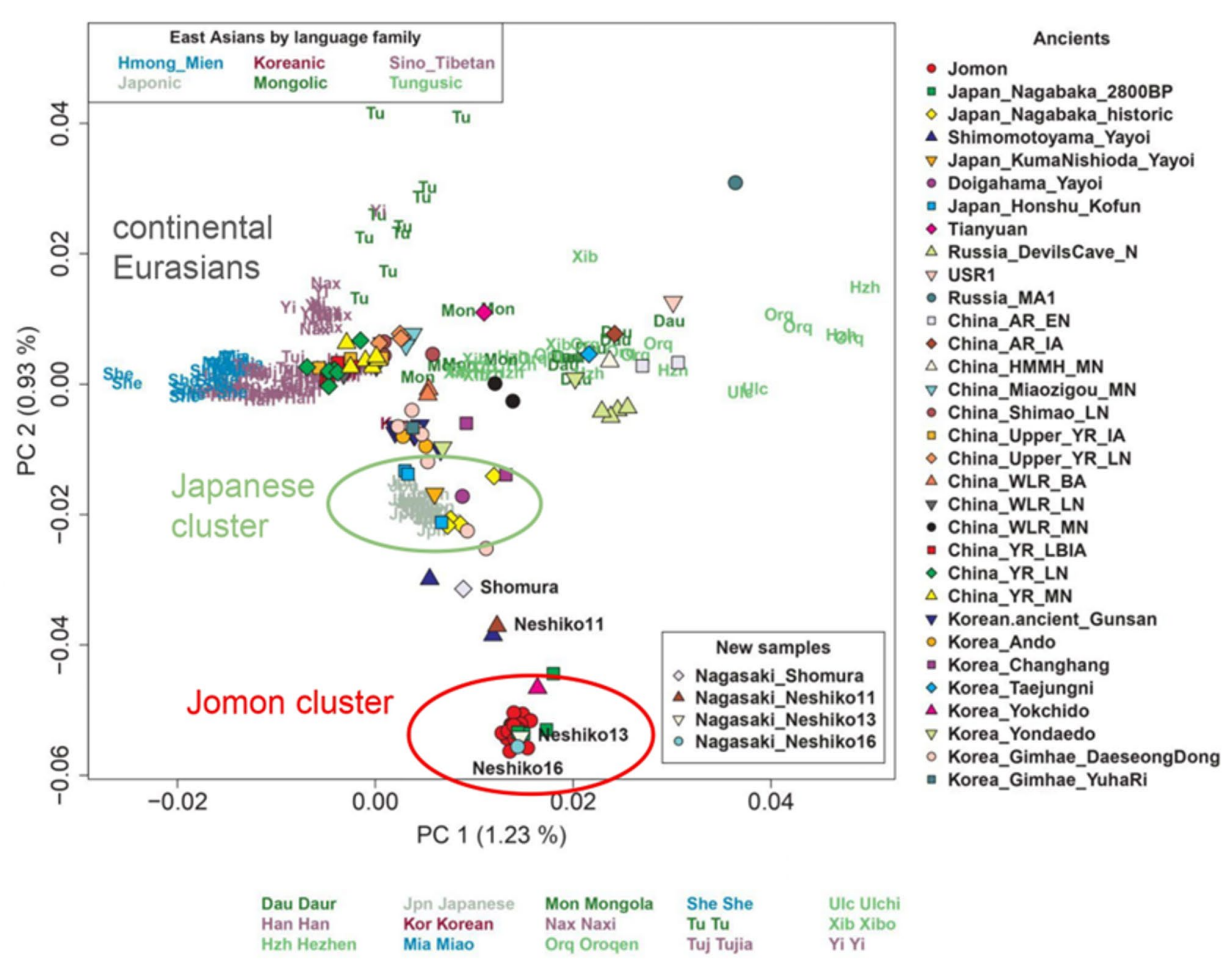
Principal component analysis of Northeast and East Asians. Modern individuals are labeled with text, whereas ancient individuals are plotted as symbols, with colors indicating population affiliation.

To investigate how Northwestern Kyushu Yayoi individuals are genetically related to other ancient and modern East Asian populations, we conducted principal component analysis (PCA) and ADMIXTURE^37^ analyses. The PCA plot shown in Fig. 2 includes data from the newly sequenced Northwestern Kyushu Yayoi genomes along with those from ancient and modern Northeast and East Asians. In this plot, PC1 appears to represent the north-south genetic cline of East Eurasians, while PC2 seems to distinguish East Eurasians from the Jomon people. At the bottom of the PCA plot, Jomon individuals from the Japanese archipelago clustered together with genetically Jomon-like individuals, including Japan_Nagabaka_2800BP from the Nagabaka site in the Ryukyu Archipelago and Korea_Yokchido from the Yokchido site, the southernmost island of Korea^38^. Present-day Japanese are positioned between the continental East Asian and Jomon clusters.

The Northwestern Kyushu Yayoi individuals (Shomura, Neshiko 11, Neshiko 13, and Neshiko 16) did not cluster with present-day Japanese; rather, they were located toward the Jomon cluster (Fig. 2). Notably, the extent of their affinity to the Jomon cluster differed among individuals. Shomura and Neshiko 11 were positioned between the Jomon and present-day Japanese clusters, similar to the Shimomotoyama Yayoi individuals, who also belong to the Northwestern Kyushu Yayoi^18^. In contrast, the two other individuals from the Neshiko site, Neshiko 13 and Neshiko 16, clustered directly with Jomon individuals.

We also confirmed that using diploid genotype data for the four Yayoi individuals yielded nearly identical PCA results (Figure S5), indicating that the overall pattern was robust to genotype calling methods. Given this consistency, we expected that analyses based on pseudo-haploid data would produce comparable results; therefore, subsequent ADMIXTURE analyses were conducted using pseudo-haploid genotypes for the four Northwestern Kyushu Yayoi individuals. A previous study showed that ancient and present-day Japanese populations can be characterized by three major ancestral components: East Asian-related, Northeast Asian-related, and Jomon-related ancestries^12^. In the ADMIXTURE analysis for *K* = 3 (Figure S6), the Shomura and Neshiko 11 individuals exhibited three major ancestry components: an East Asian-related lineage (blue), a Northeast Asian-related lineage (yellow), and a Jomon-related ancestry (red). This pattern was similar to that observed in the Northern Kyushu Yayoi individual (KumaNishioda) and the individual from the Doigahama site in the Yamaguchi Prefecture (Doigahama), as reported in a previous study^12^. These results suggest that both Shomura and Neshiko 11 were affected by admixture, carrying genetic contributions from continental ancestries while retaining a substantial proportion of Jomon-derived ancestry. In contrast, Neshiko 13 and Neshiko 16 displayed an ancestry profile indistinguishable from that of the Jomon, with no detectable contribution from either East Asian-related or Northeast Asian-related lineages, suggesting that they were likely unadmixed descendants of the Jomon people.

### Genome-wide evidence for Jomon-related ancestry

**Fig. 3:**
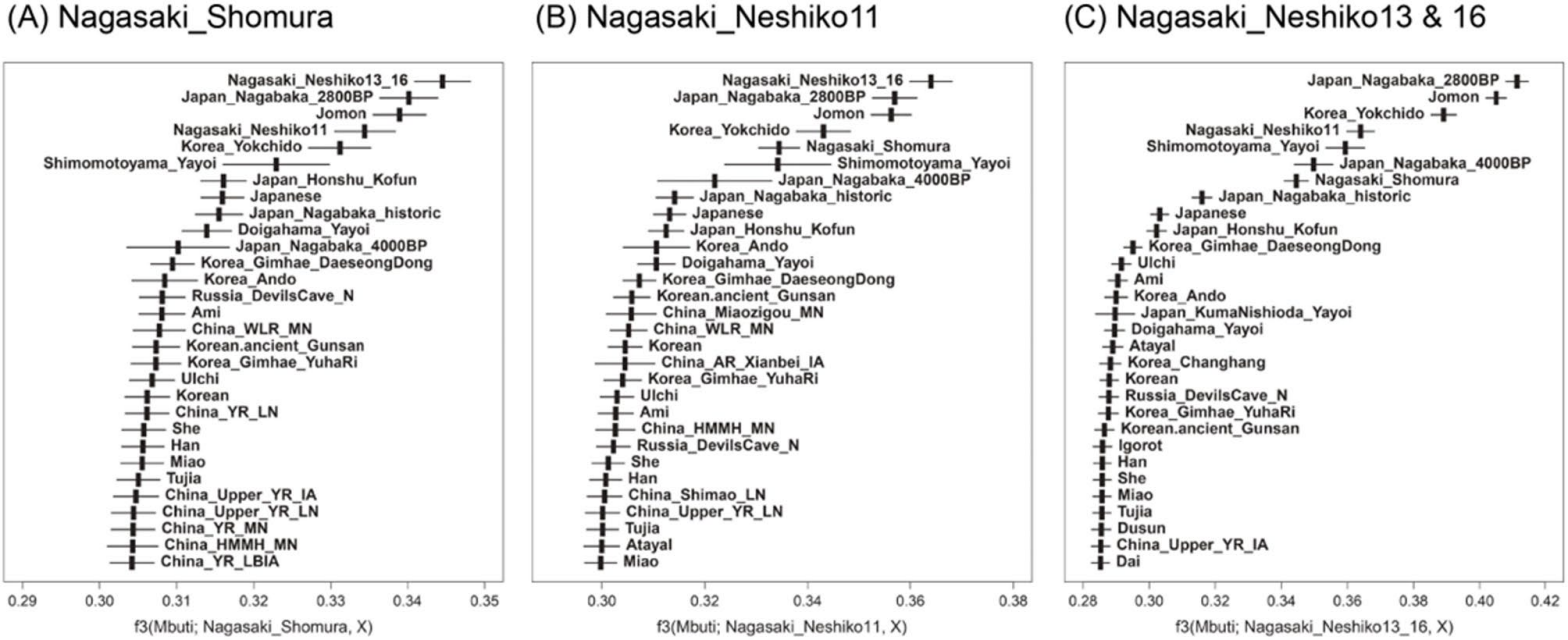
*f*3(Mbuti; target, X). Outgroup *f*3(Mbuti; target, X) was calculated for Yayoi individuals from Northwestern Kyushu, with the target set as (**A**) Shomura, (**B**) Neshiko 11, and (**C**) Neshiko 13 and Neshiko 16 (combined). For each target, the top 30 populations with the highest *f*3 values in the dataset are shown. The error bars represent the range of |Z|≤1.

To quantify the genetic affinity of Yayoi individuals to the Jomon and other populations, we performed outgroup *f*3 and *f*4 analyses. These statistics evaluate shared ancestry and help identify individuals and populations that are genetically close to the target individual or population. The outgroup *f*3-statistics of form *f*3(Mbuti, target, X), where the target corresponds to each Northwestern Kyushu Yayoi individual, showed that all four Yayoi individuals exhibited close genetic affinities with one another and consistently higher affinities to Jomon groups, including those from the Ryukyu Archipelago (Japan_Nagabaka_2800BP) and Yokchido in Korea (Korea_Yokchido)^38^, compared to present-day Japanese or any other populations (Fig. 3). Neshiko 13 and Neshiko 16 were grouped together in this analysis because they were suggested to share a close familial relationship (Table 2) and showed the highest *f*3 values (Figure S7). Neshiko 13 and Neshiko 16, which clustered with Jomon individuals in the PCA (Fig. 2) and exclusively displayed Jomon-related ancestry in the ADMIXTURE analysis (Figure S6), had a pronounced contrast in *f*3 values between the Jomon and non-Jomon populations (Fig. 3C), which is fully consistent with these patterns. Although Shomura and Neshiko 11 were inferred to have been affected by the admixture between the Jomon and continental migrant groups based on the ADMIXTURE analysis (Figure S6), both individuals were found to be genetically close to Neshiko 13 and Neshiko 16. Consistent with this interpretation, outgroup *f*3-statistics calculated using Shimomotoyama Yayoi individuals, another Northwestern Kyushu Yayoi group inferred to have been affected by admixture^18^, also showed a similarly high affinity to Neshiko 13 and Neshiko 16 (Figure S8B). Taken together, these results are most plausibly explained by the Northwestern Kyushu Yayoi individuals sharing genetic components derived from the Jomon people who lived in this region. In addition, the close genetic affinity observed between the Northwestern Kyushu Yayoi individuals and the Japan_Nagabaka_2800BP individuals (Figs. 3, S7, S8B, and S8C), which have been shown in previous studies to be genetically close to mainland Jomon populations^38^, suggests that Late Jomon people from northwestern Kyushu and the Japan_Nagabaka_2800BP individuals may have shared a relatively recent common ancestry.

Another noteworthy observation from the outgroup *f*3 analysis is that present-day Japanese individuals show a tendency to be genetically closer to Yayoi individuals who carry continental-derived ancestry (i.e., individuals such as Shomura, Neshiko 11, and the Doigahama Yayoi) than to continental populations, even though the error bars overlap and the differences are not statistically significant (Figure S8A). This trend is comparable to the affinity observed between present-day Japanese and Kofun-period individuals, indicating that both the Yayoi and Kofun groups are similar to present-day Japanese individuals. This pattern suggests that the formation of the ancestral population underlying present-day Japanese may have been underway by the Yayoi period.

**Fig. 4:**
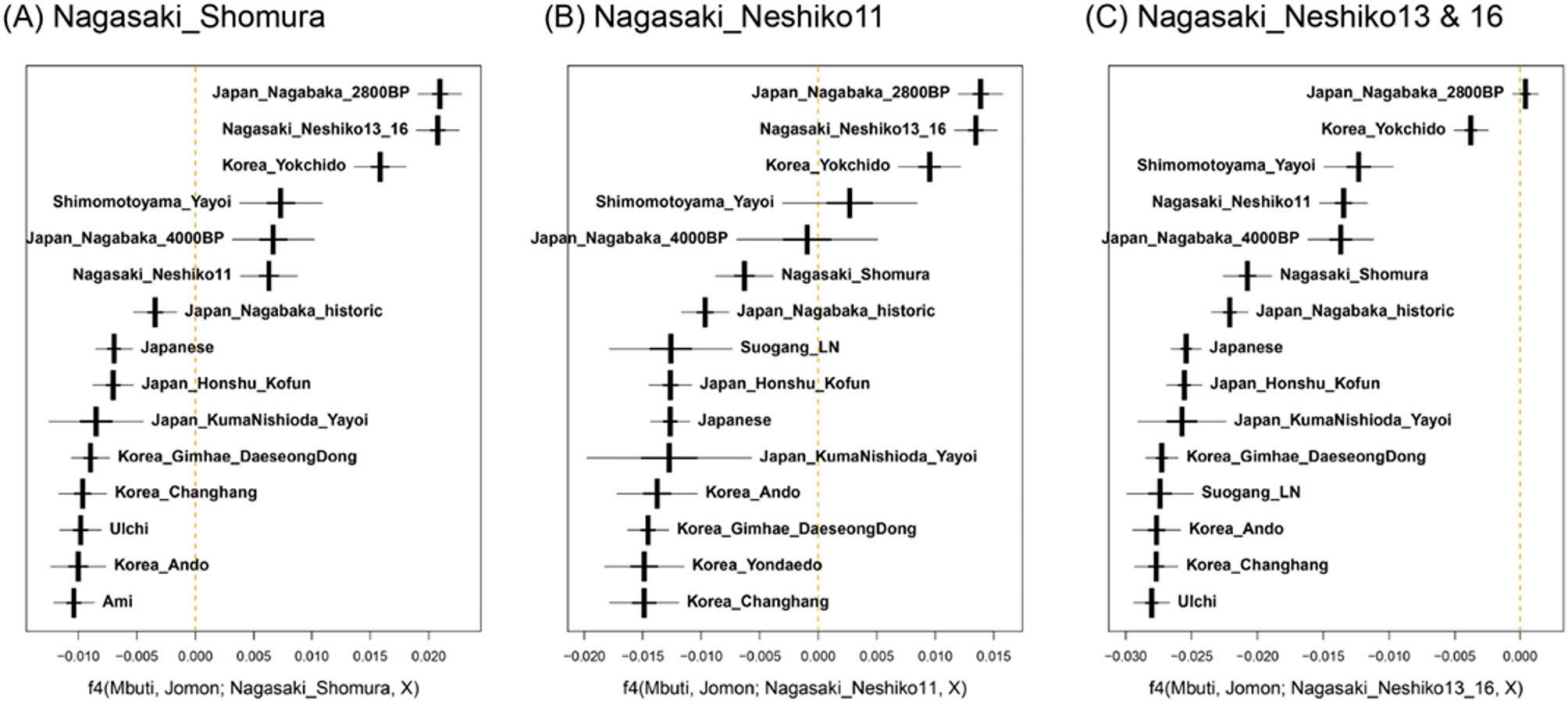
*f*4(Mbuti, Jomon; target, X). *f*4(Mbuti, Jomon; target, X) was calculated for Yayoi individuals from Northwestern Kyushu, with the target set as (**A**) Shomura, (**B**) Neshiko 11, and (**C**) Neshiko 13 and Neshiko 16 (combined). The “Jomon” population refers to previously reported Jomon genomes from mainland Japan (Honshu, Shikoku, Kyushu, and Hokkaido), which were grouped into a single population for this analysis. For each target, the top 15 populations with the highest *f*4 values in the dataset are shown. Thick error bars indicate the range of |Z|≤1, while thin error bars indicate the range of |Z|≤3.

The outgroup *f*3 analysis revealed that Shomura and Neshiko 11 are genetically close to the Jomon-like Neshiko 13 and Neshiko 16 individuals. To further evaluate which individual or population is genetically closer to each Northwestern Kyushu Yayoi individual than to the reference mainland Jomon group, we next applied *f*4-statistics of the form *f*4(Mbuti, Jomon; target, X), where “Jomon” refers specifically to the pooled mainland Jomon population (Honshu, Shikoku, Kyushu, and Hokkaido) (Fig. 4A and B). The Shomura and Neshiko 11 individuals showed significantly positive *f*4 values with Jomon-like individuals, such as Neshiko 13 and Neshiko 16 (Nagasaki_Neshiko13_16), Japan_Nagabaka_2800BP, and Korea_Yokchido, indicating that both individuals have a substantially stronger genetic affinity to these groups than to the mainland Jomon population.

In the above analyses, all four Northwestern Kyushu Yayoi individuals were suggested to be genetically close to the Jomon-like individuals from the Nagabaka site in the Ryukyu Archipelago (Japan_Nagabaka_2800BP). To evaluate whether the Late Jomon people from northwestern Kyushu and the Japan_Nagabaka_2800BP individuals shared a relatively recent common ancestry, we calculated *f*4-statistics for the Neshiko 13 and Neshiko 16 individuals, both of whom exhibited genome-wide profiles equivalent to those of the Jomon individuals (Fig. 4C). The *f*4 value being nearly zero for Japan_Nagabaka_2800BP suggests that Neshiko 13 and Neshiko 16 are at least as genetically close to Japan_Nagabaka_2800BP as they are to the mainland Jomon population.

### Regional variation in genetic affinity to Jomon populations

In the preceding *f*-statistic analyses, all mainland Jomon individuals were grouped into a single population labeled “Jomon.” However, Jomon populations are expected to exhibit regional genetic heterogeneity across the Japanese archipelago. To determine whether Northwestern Kyushu Yayoi individuals exhibit differential affinities to Jomon populations from various regions of the Japanese archipelago (Figure S9), we calculated the *f*4(Mbuti, target; Jomon1, Jomon2) statistics (Figures S10, S11, and S12). Fundamentally, the *f*4-statistic takes a negative value when the target (i.e., the Yayoi individual) is genetically closer to Jomon1 than to Jomon2 and a positive value when the target is closer to Jomon2 than to Jomon. The *f*4 analysis indicated that the Northwestern Kyushu Yayoi individuals exhibited asymmetric genetic relationships with Jomon individuals from different regions. In particular, they showed the highest genetic affinity with a Late Jomon individual from the Shikoku region, which was the Jomon sample in our dataset and was both geographically and temporally closest to the Kyushu region. This result is consistent with a previous analysis of a Yayoi individual excavated from the Doigahama site in Yamaguchi Prefecture, which is adjacent to Kyushu^12^.

Because this analysis relies on a small number of Jomon genomes from each region, and because the temporal and regional population structure of the Jomon has not yet been rigorously characterized using high-quality genomes, these results should be interpreted as preliminary. This limitation is evident for the “Japan_Shikoku_LateJomon” group, which is represented by only one low-coverage individual (< 1×)^39^, making the signal especially susceptible to stochastic variation. Even so, the emerging pattern suggests the possibility of regional differentiation among the Jomon, a hypothesis that can be more robustly evaluated as additional high-quality genomes from across the Japanese archipelago become available.

### Admixture modeling between Jomon and continental lineages

**Fig. 5:**
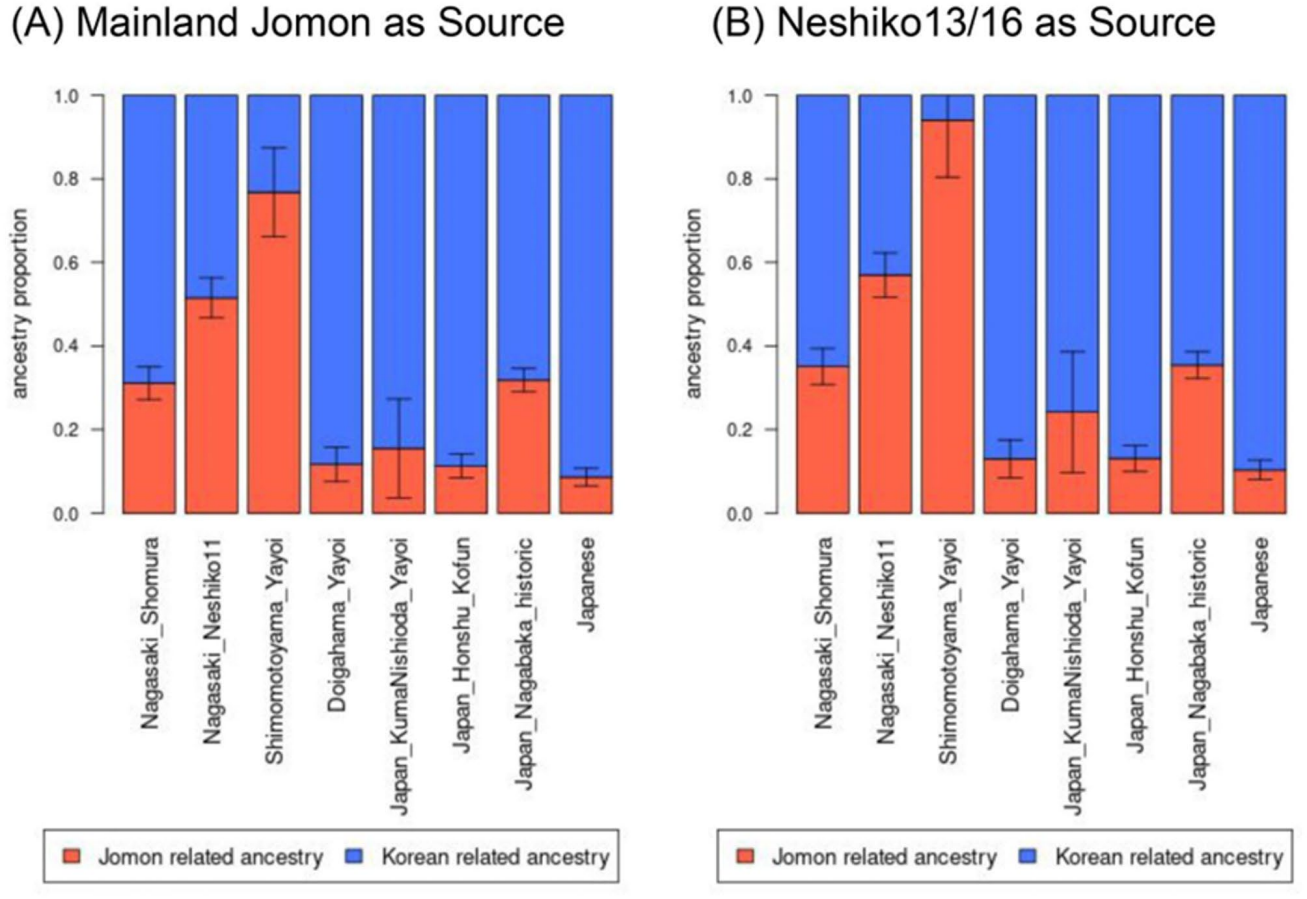
Admixture proportions estimated by qpAdm analysis. Error bars represent the range of |Z| ≤ 1. (**A**) Admixture proportions were estimated using previously reported mainland Jomons as the source of Jomon-related ancestry. (**B**) Admixture proportions were estimated using the combined data of Neshiko 13 and Neshiko 16, which were genetically classified as Jomon individuals, as sources of Jomon-related ancestry.

To model the proportion of Jomon and continental ancestry components in the Yayoi and later Japanese populations, we applied qpAdm^40^ (Fig. 5; Table S8). The analysis showed that both present-day and ancient Japanese can be modeled as deriving from an admixture between the Jomon and Korean-related lineages, with *P*-values exceeding 0.05. Furthermore, we found that, in addition to the previously published data from Jomon individuals (Fig. 5A), data from the Neshiko 13 and Neshiko 16 individuals, whose genome-wide profiles are essentially indistinguishable from those of the Jomon individuals (Figs. 2 and S6), can serve as potential sources of Jomon-related ancestry (Fig. 5B). Using Neshiko 13 and Neshiko 16 as Jomon sources led to a slight, although not statistically significant, increase in the estimated proportion of Jomon-related ancestry across the analyzed populations (Fig. 5).

Northwestern Kyushu Yayoi individuals who show evidence of mixed ancestry (i.e., Shomura, Neshiko 11, and Shimomotoyama) exhibited higher proportions of Jomon-related ancestry than Yayoi individuals from northern Kyushu (i.e., KumaNishioda Yayoi) and Yamaguchi Prefecture (i.e., Doigahama Yayoi). Despite the geographic proximity of northwestern Kyushu to the Korean Peninsula, this pattern suggests that the scale of migration into this region may have remained relatively limited up to this point in time.

### Timing of admixture events

Finally, to estimate when admixtures between Jomon- and continent-derived lineages occurred, we applied the DATES method^41^ based on the patterns of linkage disequilibrium decay. The Shomura individual was estimated to have lived approximately 4–5 generations after the admixture event (4.860 ± 1.824 or 4.222 ± 1.566 generations), whereas Neshiko 11 was estimated to have lived about 10–11 generations after the admixture (9.632 ± 2.082 or 10.602 ± 2.623 generations) (Table 3). In the DATES analysis, assuming a Jomon-Korean admixture model, the estimated admixture times for various Japanese populations ranged from approximately 2,600 to 1,900 BP. These findings are consistent with the historical view that migration from the Eurasian continent occurred gradually throughout the Yayoi period, and declined after the Kofun period.

**Table 3:** Estimated admixture dates assuming Jomon-Korean admixture models.

Target population or individual	Estimated admixture generation	Estimated admixture time (years)	Estimated radiocarbon dates	Estimated Time of Korean–Jomon Admixture
(A) Model using previously published mainland Jomon individuals as the ancestry source for the tested populations				
Shomura	4.860 ± 1.824	140.940 ± 52.896	~ 2.44 ka BP	~ 2.58 ka BP
Neshiko 11	9.632 ± 2.082	279.328 ± 60.378	~ 2.23 ka BP	~ 2.51 ka BP
Japan_Honshu_Kofun	22.868 ± 5.763	663.172 ± 167.127	~ 1.35 ka BP	~ 2.01 k BP
Japanese	66.656 ± 4.881	1933.024 ± 141.549	0	~ 1.93 ka BP
(B) Model using Neshiko13 and Neshiko 16 as the ancestry source for the tested populations				
Shomura	4.222 ± 1.566	122.438 ± 45.414	~ 2.44 ka BP	~ 2.56 ka BP
Neshiko 11	10.602 ± 2.623	307.458 ± 76.067	~ 2.23 ka BP	~ 2.54 ka BP
Japan_Honshu_Kofun	24.053 ± 7.657	697.537 ± 222.053	~ 1.35 ka BP	~ 2.05 ka BP
Japanese	72.032 ± 9.179	2088.928 ± 266.191	0	~ 2.09 ka BP

## Discussion

The ancient genomes of Northwestern Kyushu Yayoi excavated in Nagasaki Prefecture exhibited significantly higher proportions of Jomon-derived ancestry than present-day Japanese populations, and the individuals themselves showed substantial variation in the proportions of this ancestry. The results of the admixture dating further provided important insights into the timing of migration from the Korean Peninsula to the Japanese archipelago.

The beginning of the Yayoi period was defined primarily based on archaeological criteria, such as the appearance of distinctive Yayoi pottery styles characterized by burnished reddish surfaces and sunken patterns (incised line decorations), wet rice agriculture, and other cultural elements, and remains a subject of scholarly debate, with estimates ranging from the 10th to the 3rd century BCE. Recent radiocarbon dating of charred botanical remains and other archaeological materials has suggested that the onset of the Yayoi period dates back as early as the 10th to 9th centuries BCE^42,43^. While such chronologies reflect cultural changes, they do not directly reveal when biological interactions such as admixtures between populations began. In this regard, our study adds a genetic perspective by estimating the timing of admixture between the Jomon people and migrants from the Korean Peninsula. Specifically, admixture dating of the ~ 2,441-year-old Shomura individual suggests that gene flow had already begun in this region just under 2.6 kya. Although this genetic evidence does not redefine the beginning of the Yayoi period, it provides additional insight into the timing of demographic interactions that likely accompanied the associated cultural transformations.

Among ancient Japanese genomes from the post-Yayoi period, the Ikawazu individual (IK002), dated to approximately 2,681 years before present^4^, uniquely displays a genetic profile that is largely consistent with that of the Jomon. In this study, we identified two Northwestern Kyushu Yayoi individuals, Neshiko 13 and Neshiko 16, dated to 2,340 and 2,355 years ago, whose genomes are composed almost entirely of Jomon ancestry. These findings demonstrate that during this period, the Japanese archipelago was inhabited not only by individuals of mixed ancestry between the Jomon and continental migrants but also by people who were direct descendants of the Jomon. Notably, such Jomon-descendant individuals were present even in northwestern Kyushu, a region geographically closest to the Korean Peninsula and regarded as a primary entry point for continental migrants. This situation parallels other cases worldwide in which indigenous genetic lineages persist locally despite major cultural and demographic transitions. For example, the Mesolithic hunter-gatherer ancestry remained in parts of Iberia during the Neolithic, and a similar persistence of forager ancestry was observed in Scandinavia and sub-Saharan Africa^44–46^. Such examples highlight that cultural transitions do not necessarily entail complete genetic replacement but can result in complex mosaics of continuity and admixture. In Japan, this meant that during the Middle Yayoi period, populations included both admixed descendants of continental migrants and direct Jomon lineages, representing one of the most genetically diverse moments in Japanese prehistory.

Previous genetic studies have shown that after the Yayoi period, the admixture between the Jomon and continental-derived populations progressed throughout much of mainland Japan. Although present-day populations in the Tohoku region retain slightly higher proportions of the Jomon ancestry, the overall ratio of Jomon to continental ancestry is remarkably uniform among mainland Japanese^47^. In contrast, peripheral groups, such as the Ainu and Ryukyuans, appear to have been less affected by continental migrants. Genome-wide analyses have shown that they form distinct genetic clusters and retain higher proportions of Jomon ancestry than mainland Japanese^48^. These broader patterns provide an important contextual framework for interpreting our findings in northwestern Kyushu, which captures one of the early local stages of this demographic transition.

Our study revealed substantial genetic diversity within the Northwestern Kyushu Yayoi, a group previously defined largely on the basis of shared Jomon-like morphological traits. The wide variation in Jomon ancestry observed among individuals from the Shomura, Neshiko, and Shimomotoyama sites indicates that the Northwestern Kyushu Yayoi cannot be regarded as a homogeneous population. Although the individuals analyzed here may not fully capture the genetic diversity of the entire population, increasing the number of samples in future studies will be essential for refining our understanding of the genetic landscape of the Yayoi people in this region.

## Materials and methods

### Ancient sample information

In this study, we first conducted radiocarbon dating of bone collagen extracted from four Northwestern Kyushu Yayoi individuals excavated from two Middle Yayoi period sites in Nagasaki Prefecture, Japan, to confirm their chronological placement. One adult male (Shomura) was recovered from a stone coffin at the Shomura site on Iki Island^19^. Although his humerus and femur were well preserved, most of the skull was missing, precluding morphological determination. In addition, three individuals were excavated from the Neshiko site, a Middle Yayoi period settlement on Hirado Island^20,21^: one adult male (Neshiko 11), one child (Neshiko 13), and one adult female (Neshiko 16). Following radiocarbon dating, all four individuals were sequenced for population genomic analyses. This study was approved by the Ethics Committee of Toho University School of Medicine (A23103_A20110_A18099_A18056).

### Radiocarbon dating and stable isotope analysis

Each bone fragment was initially cleaned by sandblasting to remove surface contaminants, such as adhering sediments. This was followed by an ultrasonic treatment in deionized water and an 8-hour immersion in acetone. To further eliminate humic substances, the samples were soaked in 0.2 M sodium hydroxide (NaOH) solution for 8 h. After thorough rinsing and drying, collagen was extracted from the treated bone material according to established protocols. Radiocarbon dating of the extracted collagen was conducted using a compact accelerator mass spectrometry (AMS) system equipped with a 0.5 MV Pelletron accelerator (National Electrostatics Corporation, Middleton, WI, USA). The measurements were performed at the Yamagata University AMS facility (YU-AMS), Japan^49^. The obtained radiocarbon ages were calibrated using OxCal version 4.4^50^. Stable carbon and nitrogen isotope ratios in collagen were analyzed using an elemental analyzer (EA; Vario MICRO cube, Elementar Analysensysteme GmbH, Langenselbold, Hesse, Germany) coupled with an isotope ratio mass spectrometer (IRMS; IsoPrime, Isoprime Ltd., Manchester, UK). Both instruments were housed at the YU-AMS facility^51^. The carbon (^13^C/^12^C) and nitrogen (^15^N/^14^N) isotopic values are expressed as δ^13^C and δ^15^N relative to internationally accepted standards.

### DNA extraction

DNA was extracted from the petrous bones of four individuals: Shomura, Neshiko 11, Neshiko 13, and Neshiko 16. The left petrous bone was used for Shomura and Neshiko 11 and the right petrous bone was used for Neshiko 13 and Neshiko 16 (Figure S1). The sampling targeted the dense region of the petrous bone surrounding the cochlea. The outer surface of each bone was removed using a sanding tool (Dremel, Robert Bosch Tool Corporation, Mt. Prospect, IL, USA) and the cleaned inner portion was ground into a fine powder using a bead mill (Multi-Beads Shocker MB601U; Yasui Kikai, Osaka, Japan). The powder was decalcified in 0.5 M EDTA (pH 8.0) at 56 °C for 2 h in a rotating oven. After centrifugation, the supernatant was discarded, and decalcification was repeated three times. DNA was extracted using phenol: chloroform: isoamyl alcohol (25:24:1), followed by additional chloroform extraction. The aqueous phase was recovered, concentrated to 200 µL using Amicon Ultra-15 centrifugal filters (Merck Millipore, Darmstadt, Germany), and further purified using a MiniElute silica spin column (QIAGEN, Hilden, Germany).

### Library preparation and target enrichment

Next-generation sequencing libraries were constructed as previously described^52,53^. For the Shomura individual, both double-stranded (DS) and single-stranded (SS) libraries were prepared and used for shotgun sequencing. In contrast, due to poor DNA preservation, only SS libraries were constructed for the three Neshiko individuals (Neshiko 11, Neshiko 13, and Neshiko 16). These libraries were subjected to in-solution nuclear DNA target enrichment using a Twist Ancient DNA Panel (Twist Bioscience, South San Francisco, CA, USA), which captures approximately 1.2 million genome-wide single nucleotide polymorphisms (SNPs). Enrichment was performed according to the manufacturer’s protocol and the procedures described by Rohland et al.^54^ using the Twist Hybridization and Wash Kit.

### Sequencing and data processing

Enriched libraries were amplified and sequenced on an Illumina MiSeq platform (150-cycle v3 kit) for preliminary evaluation. Based on the assessment of DNA preservation and authenticity, high-coverage sequencing was conducted on the Illumina NovaSeq 6000 platform (150 bp paired-end reads) at SRL Inc. (Tokyo, Japan). Initially, Illumina adapter sequences were trimmed from the raw FASTQ files using AdapterRemoval v2.3.0^55^, with filters applied to exclude reads shorter than 30 base pairs and to remove low-quality bases at the ends of the reads using the following parameters: ‘--trimns --trimqualities --minlength 30 --minquality 20 --minadapteroverlap 1 --collapse’. For SS libraries, the parameter ‘--trim5p 0 15’ was additionally used to remove the low-diversity tail at the 5′ end of R2 reads, based on quality assessments conducted using fastQC v0.11.9^56^. Following this, the well-merged, adapter-trimmed reads were mapped to the human reference genome hs37d5 using the aln and samse modules in Burrows-Wheeler Aligner (BWA) v0.7.17^57^, with the following parameters: ‘-l 16500 -n 0.01 -o 2’. The output BAM files were sorted, and reads with a Phred-scaled mapping quality score below 30 were discarded using SAMtools v1.19^58^. PCR duplicates were subsequently removed using the MarkDuplicates tool from Picard v3.1.1 (https://broadinstitute.github.io/picard/*).* Ancient DNA damage patterns were assessed using mapDamage v2.2.2^59^ (Figure S4). Based on the mapDamage plots, 10 bp were clipped from both ends of all reads in the DS library data and 3–4 bp were clipped from both ends of all reads in the SS library data using the trimBam function on bamUtils v1.0.15^60^ to minimize the impact of ancient DNA damage.

### Authentication and contamination estimates

Modern DNA contamination was assessed using various methods. For male individuals, X chromosome–based contamination was estimated with ANGSD v0.941^61^ using both the ‘MoM’ and ‘new_llh’ approaches. Autosomal contamination was evaluated using verifyBamID v2.0.1^62^ with the 1000 Genomes 100 K SNP reference panel. All the estimates indicated low contamination levels (Table S2). After verification, the DS and SS BAM files of the Shomura individual were merged using SAMtools v1.19^58^. Contamination was reassessed for the merged files to ensure consistency across libraries. The mean genome-wide coverage of the Shomura individual was computed using Qualimap v2.3^63^ (Table S3).

### Genotype calling and filtering

Diploid genotypes for the newly sequenced target individuals were called for each locus using the Genome Analysis Toolkit (GATK) v3.8^64^. A GVCF file was generated using the HaplotypeCaller module with the options ‘-ERC GVCF -rf BadCigar -mbq 30’, and a VCF file was then produced from the GVCF file using the GenotypeGVCFs module with the ‘-allSites’ option. Variant sites were filtered using BCFtools v1.20^58^, retaining only those with a minimum depth of 10 and both a QUAL score and a genotype quality score (GQ or RGQ) of at least 30 on the Phred scale. The final number of obtained 1240K autosomal SNPs is presented in Table S4.

Pseudo-haploid genotypes were also generated by randomly sampling a single high-quality base for each autosomal SNP in the 1240 K panel, using SAMtools mpileup with the ‘-R -B -q30 -Q30’ options, followed by the randomHaploid mode in pileupCaller v1.5.2 (https://github.com/stschiff/sequenceTools). In this study, pseudo-haploid data were used only for the PCA (Figure S5) and the ADMIXTURE analysis (Figure S6). The quality metrics for the 1240 K autosomal SNPs for each sample are presented in Table S5.

### Sex identification

The sex of each sample was estimated based on the number of reads mapped to the X and Y chromosomes (Table S6). The read counts for each chromosome were computed using the ‘idxstats’ command in SAMtools v1.19^58^. Based on these results, the $\:{R}_{Y}$ index, Y/(X + Y), of each sample was calculated, and the genetic sex was determined following the method described in a previous study^65^.

### MtDNA and Y chromosome haplogroup

For mtDNA and Y chromosome haplogroup analysis, mitochondrial and Y chromosomal sites were extracted with ‘FORMAT/DP > = 1’ from non–quality-filtered VCF files using BCFtools v1.20. The mtDNA haplogroup was determined using Haplogrep v3.2.1^66^ and the Y-chromosome haplogroup was identified using Y-LineageTracker v1.3.0^67^.

### Dataset compilation

For population genetic analysis, the newly sequenced ancient Yayoi genomes were merged with the 1240 K SNP dataset (v54.1.p1), which was downloaded from the Allen Ancient DNA Resource (AADR)^68^ (https://reich.hms.harvard.edu/allen-ancient-dna-resource-aadr-downloadable-genotypes-present-day-and-ancient-dna-data), and processed in the same manner as described in our previous study^12^. Human Genome Diversity Project (HGDP) data^69^ and Simons Genome Diversity Project (SGDP) data^70^ were extracted from modern population panels. One individual from each pair with a KING kinship coefficient greater than 0.0884^36^ was excluded using PLINK v2.00a5^71^. The China_Lahu outlier (HGDP01319) was also excluded based on our previous analysis. In addition, ancient Eurasian genomes^4,38,39,41,72–80^ were extracted from the 1240 K dataset, and additional ancient genomes from Japan and Korea^3,12,18,81^ were merged.

### Genetic kinship analysis

Kinship coefficients (KING-robust^36^ were calculated for all six possible pairs among the four Yayoi individuals, based on 1,150,639 autosomal SNPs, using PLINK v2.00a^71^. As no second-degree familial relationships were detected, all four Yayoi individuals were included in the subsequent population genetic analyses. To further assess possible third- to sixth-degree familial relationships, identity-by-descent (IBD) was estimated using TRUFFLE v1.38 program^82^.

### Population clustering analyses

Principal component analysis (PCA) was conducted using smartpca v16000 from the EIGENSOFT package v7.2.1^83^. Ancient genomes were projected onto the PC space calculated from modern individuals using the ‘lsqproject: YES’ option. Additionally, the ‘shrinkmode: YES’ option was applied when the present-day population set included only East Eurasian populations.

The ADMIXTURE v1.3.0 program^40^ was used for unsupervised model-based genetic clustering. To avoid biases arising from the coexistence of diploid and haploid genomes, pseudo-haploid-called modern individuals from the 1000 Genomes Project^84^, as available in the AADR 1240 K SNP dataset, were used instead of the diploid-called HGDP and SGDP data. Five East Asian populations (CDX, CHB, CHS, KHV, and JPT) from the 1000 Genomes Project were selected as modern reference panels.

Before running ADMIXTURE, the absence of bias between the pseudo-haploid and diploid datasets was confirmed by principal component analysis (Figure S5). The haploid genotype dataset was then merged, and SNPs with a minor allele frequency below 0.01 were filtered out using PLINK v1.90b^85^ with the ‘--maf 0.01’ option. Linkage disequilibrium (LD) pruning was performed using PLINK v1.9 with the ‘--indep-pairwise 200 25 0.2’ option, applying a window size of 200 SNPs, a step size of 25 SNPs, and an *r*² threshold of 0.2. This process retained 368,630 SNPs. ADMIXTURE was run with the ‘--haploid=‘*’ --cv’ options.

### *f*-statistics

To assess the genetic relationships between the target ancient and other East Eurasian populations, we computed the *f*-statistics using the qp3Pop v651 and qpDstat v980 programs in the ADMIXTOOLS v7.0.2 package^40^.

First, outgroup *f*3-statistics of the form *f*3(Mbuti; target, X) were calculated, where Mbuti pygmies served as the outgroup and X represented one of the populations in the dataset. In addition, *f*4-statistics of the form *f*4(Mbuti, Jomon; target, X) were computed to identify the Jomon population that was genetically closer to each Northwestern Kyushu Yayoi individual than to the reference Jomon group from mainland Japan. Here, previously reported Jomon genomes from mainland Japan (Honshu, Shikoku, Kyushu, and Hokkaido) were grouped into a single reference population labeled “Jomon.” For all *f*-statistics, *Z*-scores representing the deviation from zero in units of standard error were also calculated.

Furthermore, *f*4-statistics of the form *f*4(Mbuti, target; Jomon1, Jomon2) were calculated to test whether any Jomon subpopulations exhibited exceptionally high genetic affinity for the target individuals. The archaeological locations of the Jomon groups used in this analysis are shown in Figure S9.

### Admixture modeling

The qpWave and qpAdm v1520 programs in the ADMIXTOOLS v7.0.2 package were used to model newly sequenced ancient individuals as an admixture of Jomon and continental migrants. These programs were run without the “allsnps: YES” option to avoid potential bias introduced by the option^86^.

The following populations were used as outgroup references for qpAdm modeling: Mbuti, Italy_North_Villabruna_HG^73^, Iran_GanjDareh_N^41^, Tianyuan^74^, USR1^75^, Papuan, Ami, Bianbian_EN, Liangdao2_EN^78^, and She.

The qpWave program was run to assess the independence of the outgroup populations using a significance threshold of *P* = 0.01. After confirming the independence of the outgroups, a two-way admixture model involving Jomon and present-day Koreans (from the SGDP dataset) was tested for both ancient and present-day Japanese target populations using qpAdm, in accordance with our previous findings^12^. Two Jomon–Korean admixture models were tested: one using mainland Jomon genomes (Honshu, Shikoku, Kyushu, and Hokkaido) grouped into a single population, and the other using the combined Neshiko 13 and Neshiko 16 data as the Jomon-related source. All qpAdm models were computed using more than 50,000 SNPs after dataset intersection; therefore, we considered the results to be reliable without applying an explicit minimum SNP threshold.

### Estimation of admixture date

Admixture dates were estimated for the Yayoi individuals from the Shomura and Neshiko sites, as well as for present-day Japanese and Kofun-period Japanese, using DATES v753^41^ with the following parameters: “jackknife: YES, maxdis: 1.0, binsize: 0.001, runmode: 1, mincount: 1, runfit: YES, lovalfit: 0.45, afffit: YES, and qbin: 100”. Two Jomon–Korean admixture models were examined: one using mainland Jomon genomes as the Jomon source and another using the combined Neshiko 13 and Neshiko 16 data as the Jomon-related source. A generation time of 29 years, which is the default value in the DATES program, was assumed to convert the number of generations into calendar years. The estimated radiocarbon dates for Japan_Honshu_Kofun were calculated as the average of those of the three individuals: 1378 BP, 1340 BP, and 1325 BP.

## Supplementary Information

Below is the link to the electronic supplementary material.

Supplementary Material 1

Supplementary Material 2

Supplementary Material 3

Supplementary Material 4

## Data Availability

The fastq-format raw sequencing reads and BAM-format genome files of Shomura, Neshiko 11, Neshiko 13, and Neshiko 16 were deposited in the DDBJ Sequence Read Archive under the BioProject accession number PRJDB35646 (https://ddbj.nig.ac.jp/resource/bioproject/PRJDB35646).
