# Supplementary figures and images for: Ancient genomes reveal early-stage admixture and genetic diversity in the Northwestern Kyushu Yayoi
Source: Sci Rep. 2026 Jan 7;16:4833. doi: 10.1038/s41598-026-34996-7 (PMC12873284; doi:10.1038/s41598-026-34996-7)

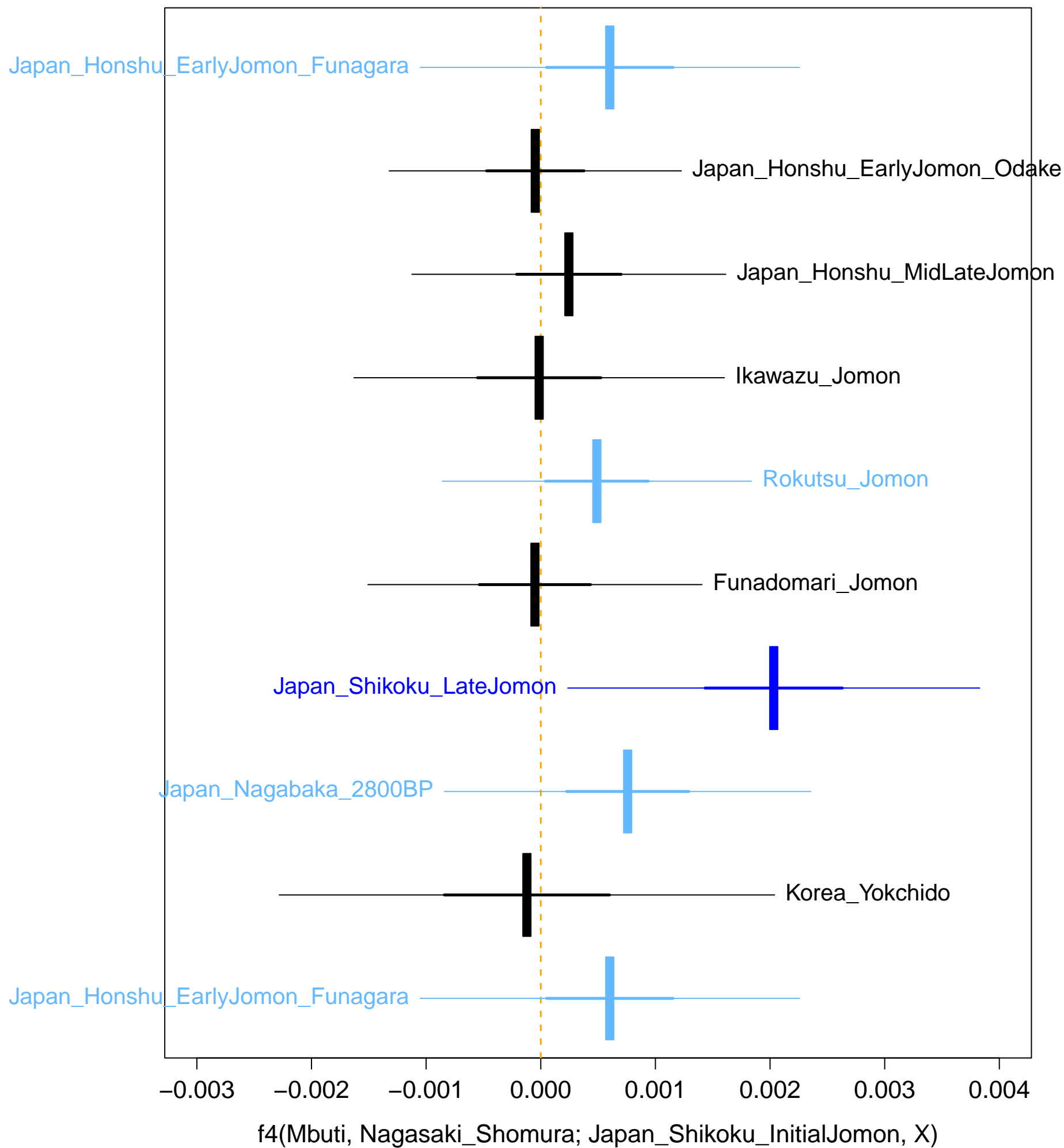

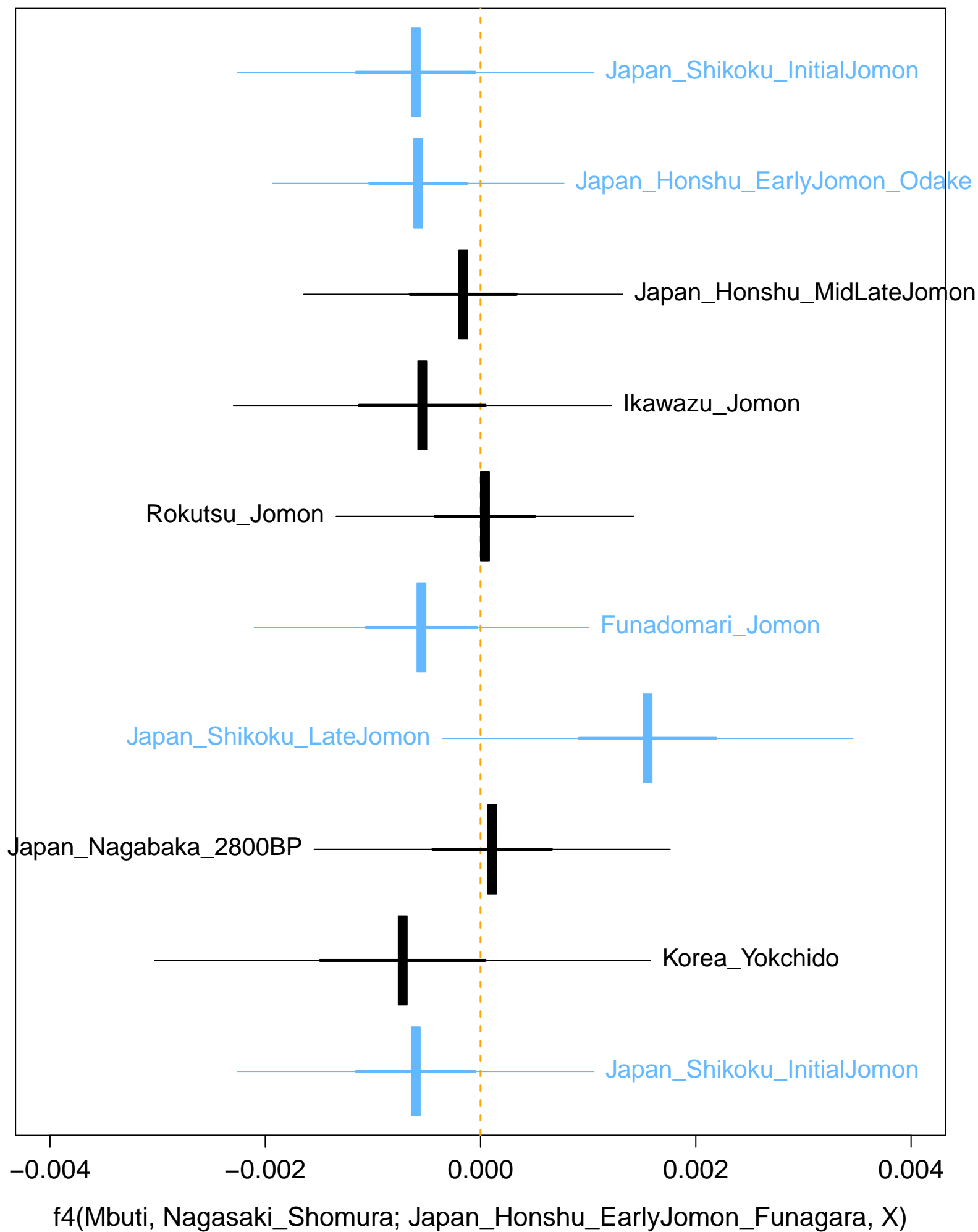

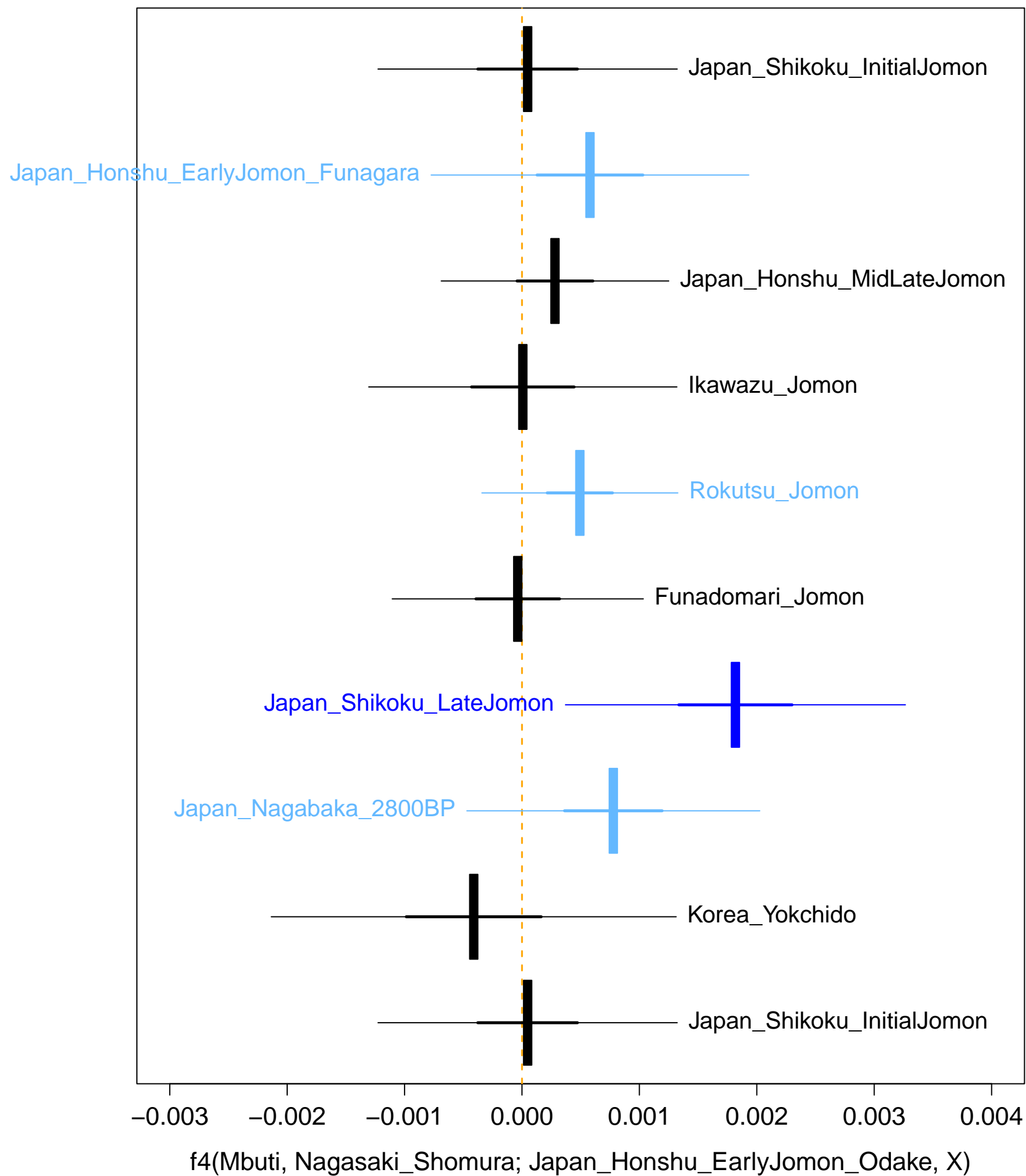

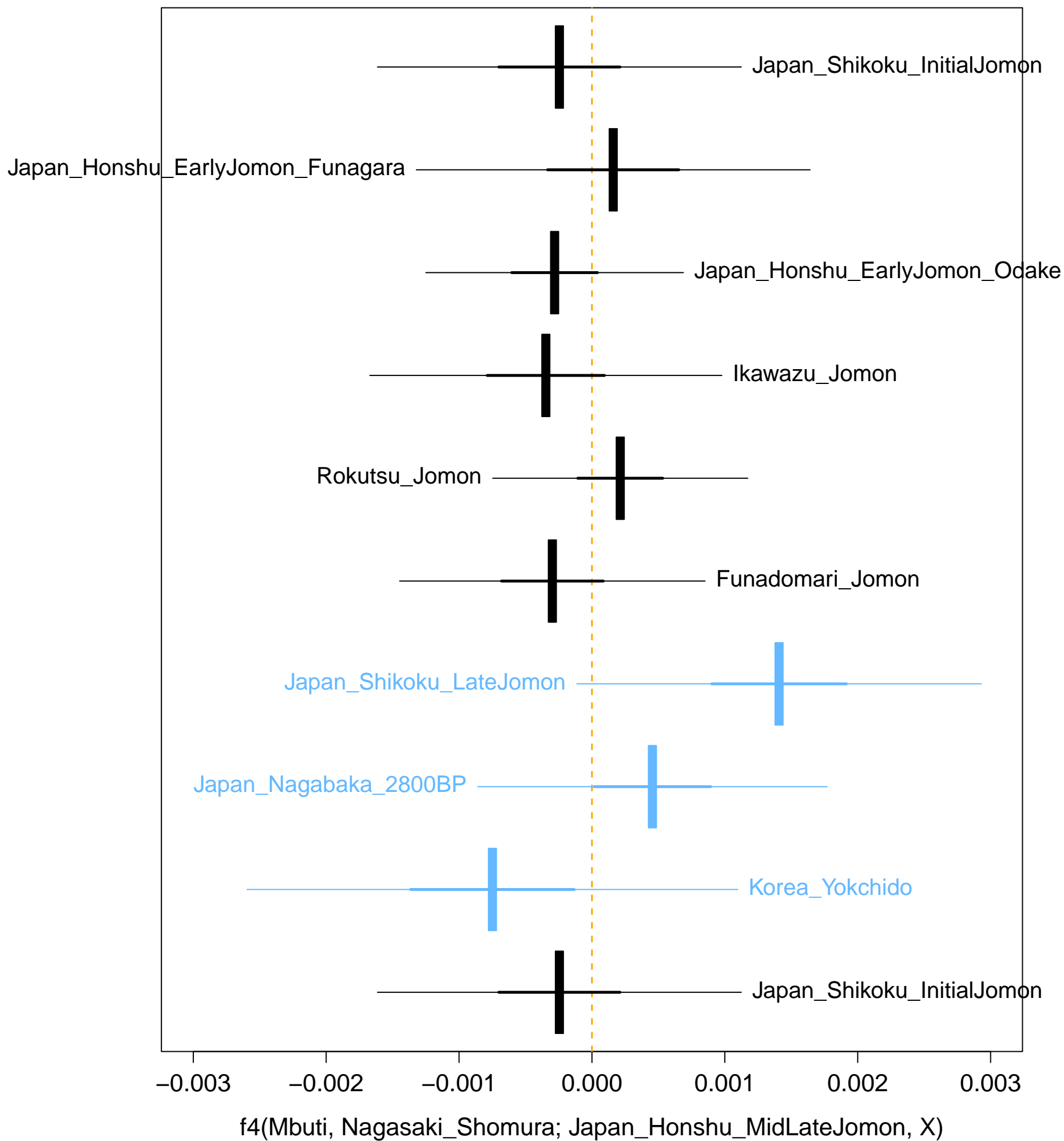

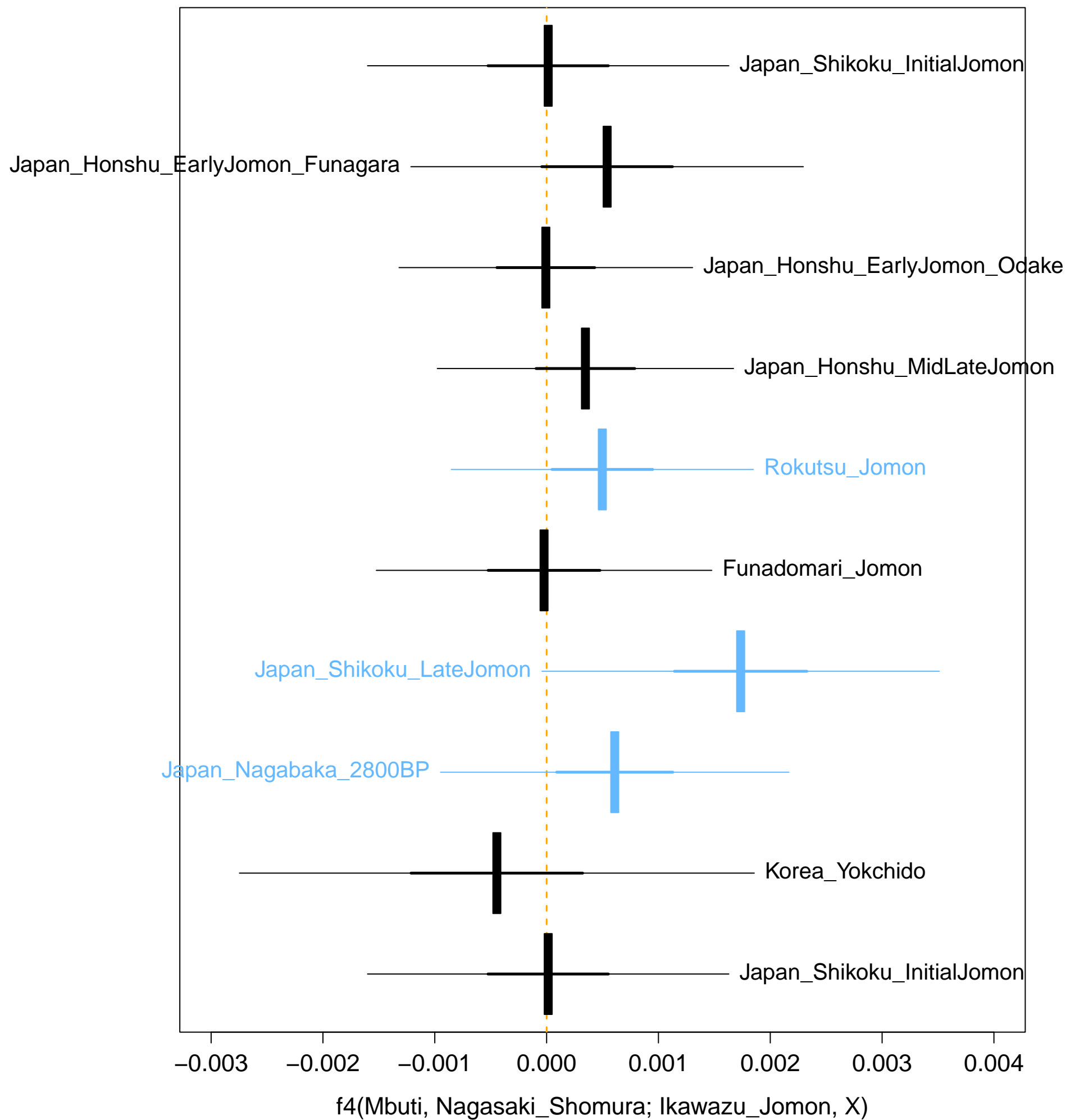

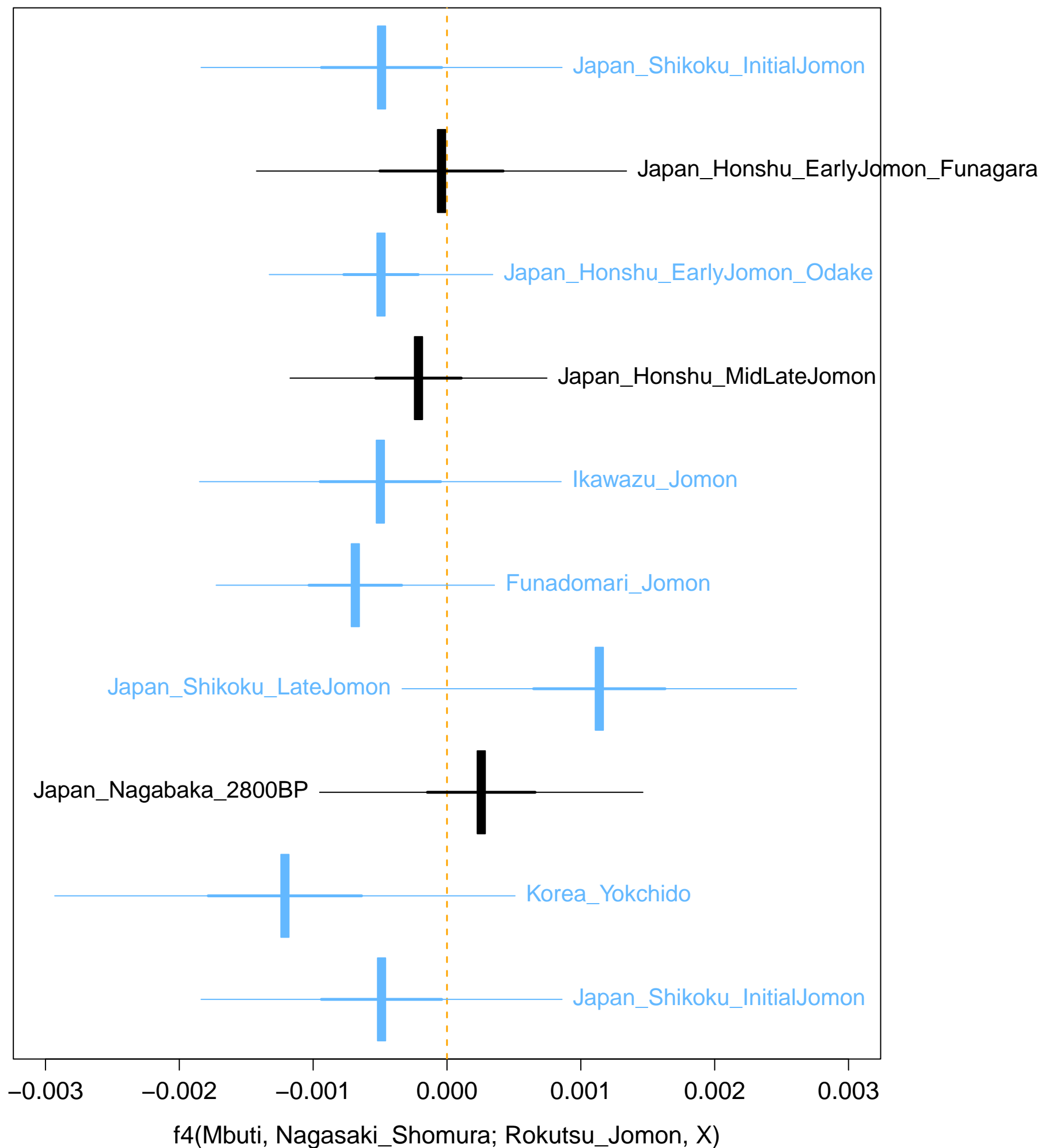

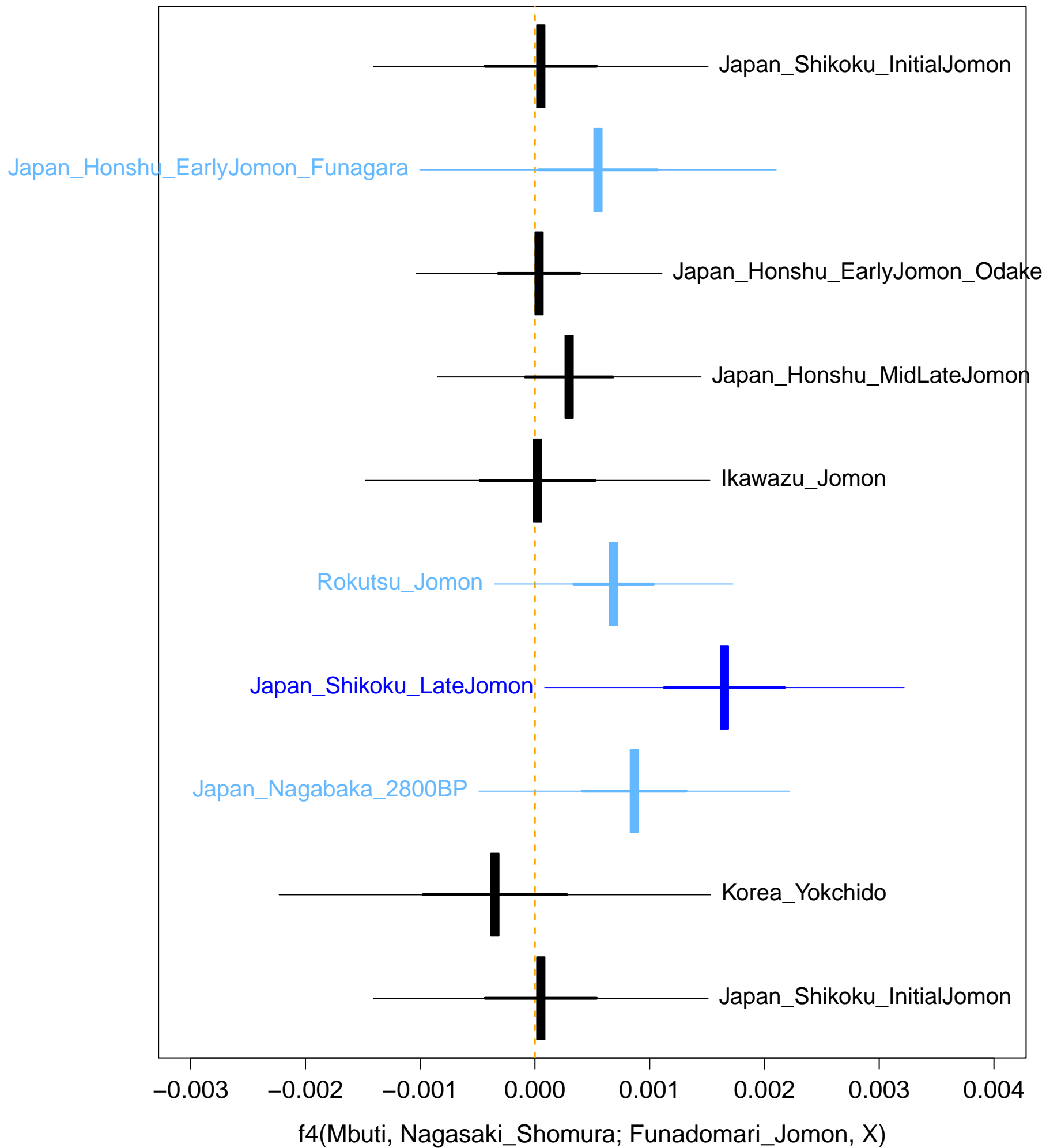

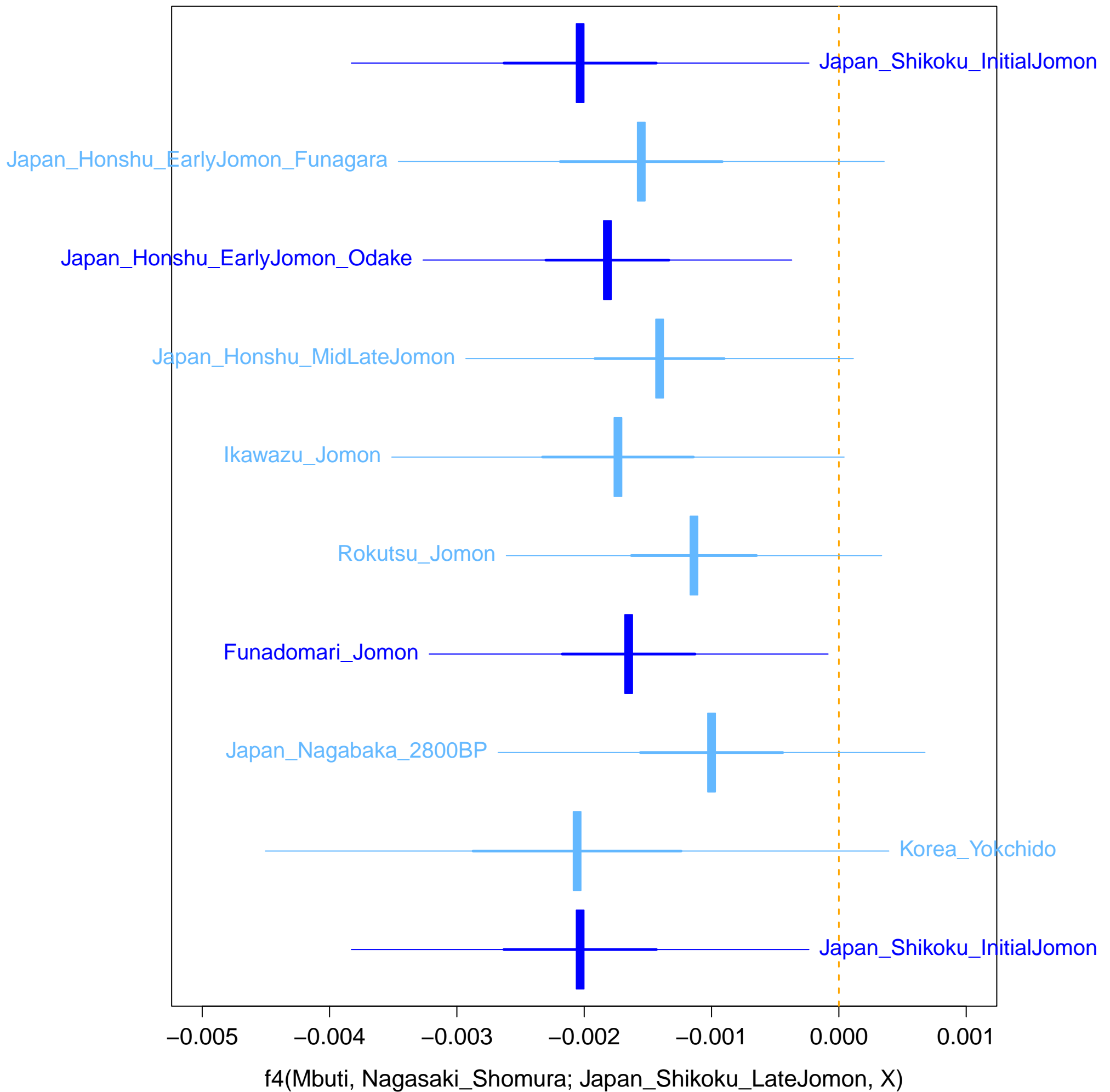

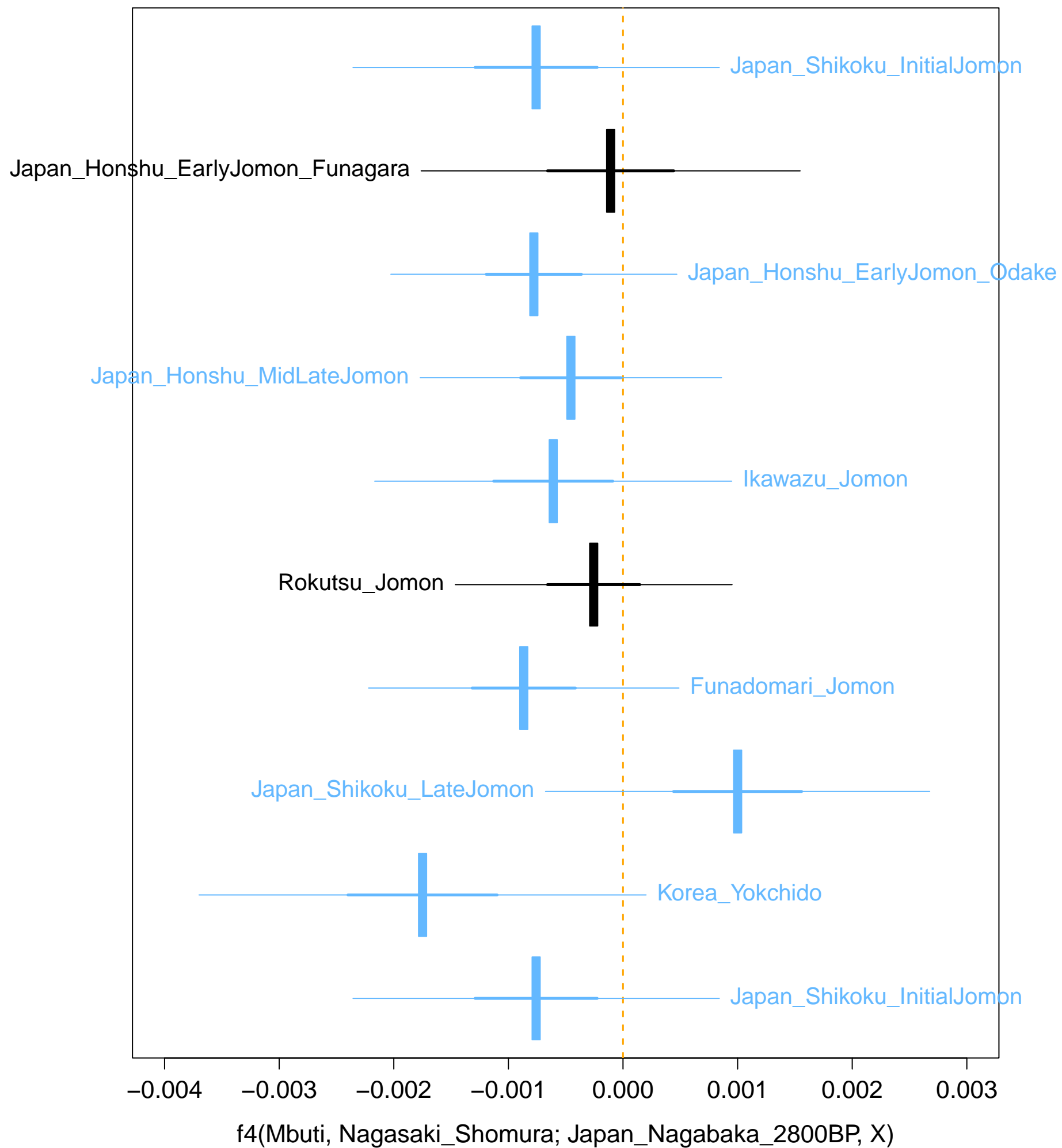

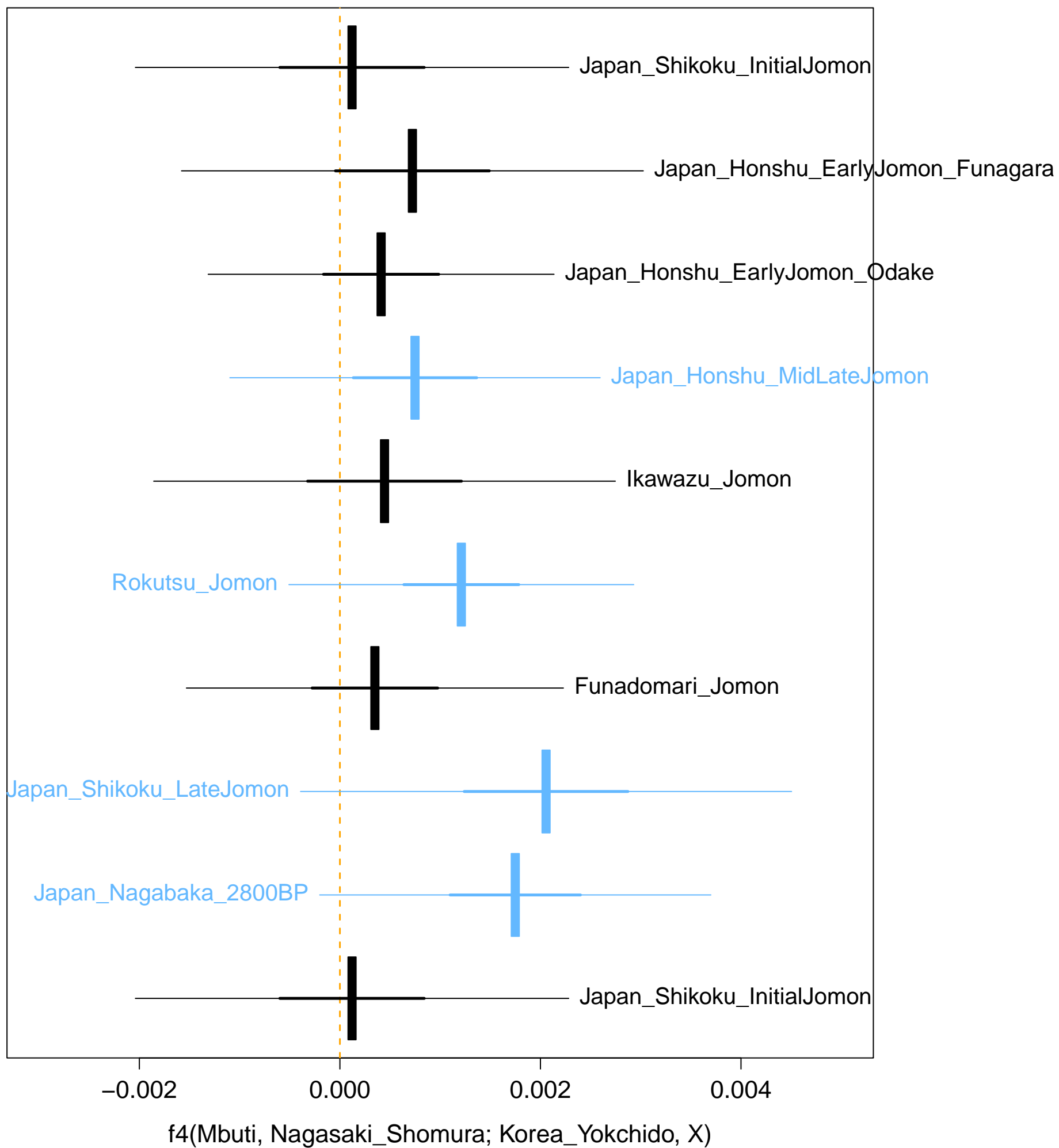

Supplement: Supplementary file 1 — Supplementary Material 1 [file 41598_2026_34996_MOESM1_ESM.pdf]

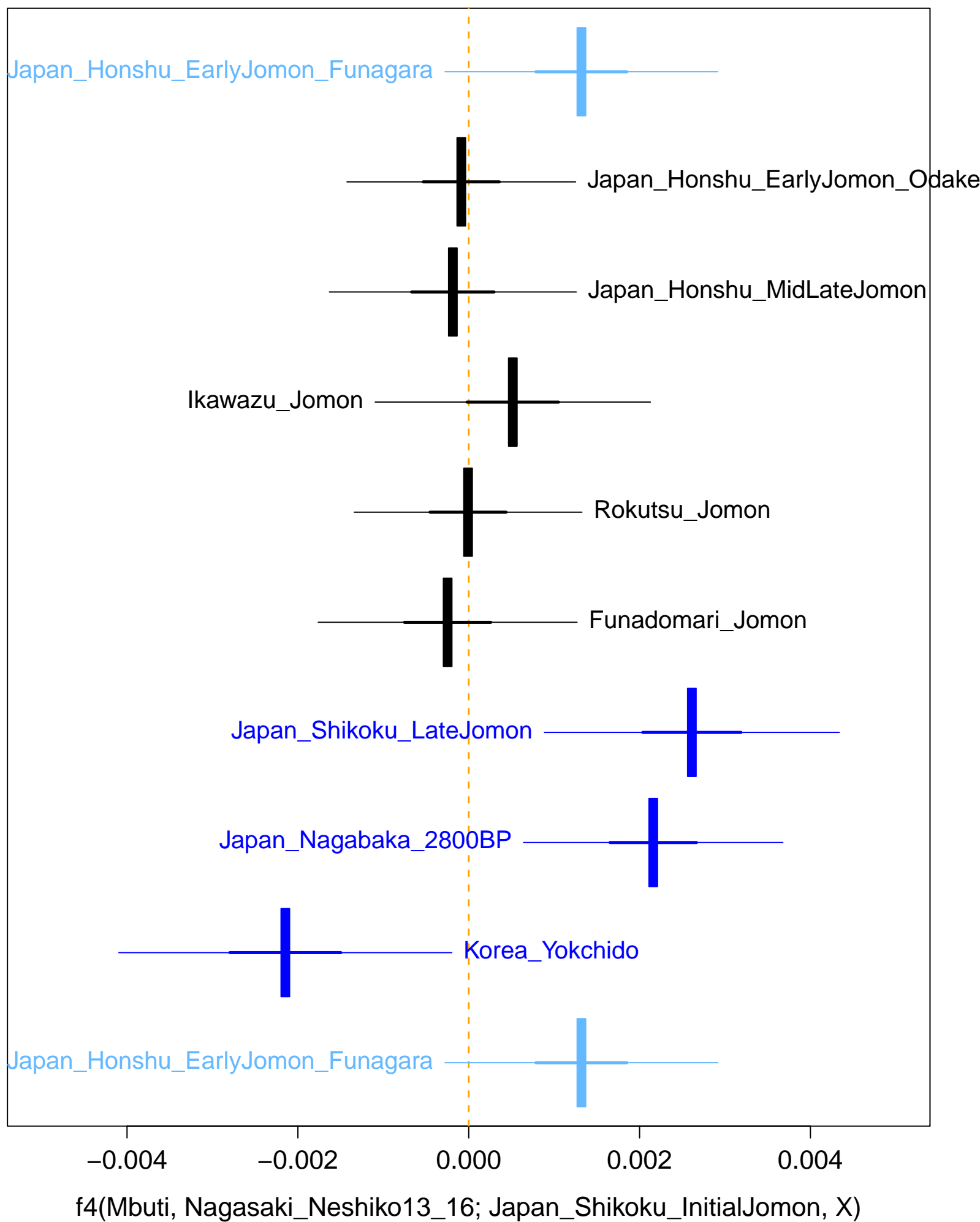

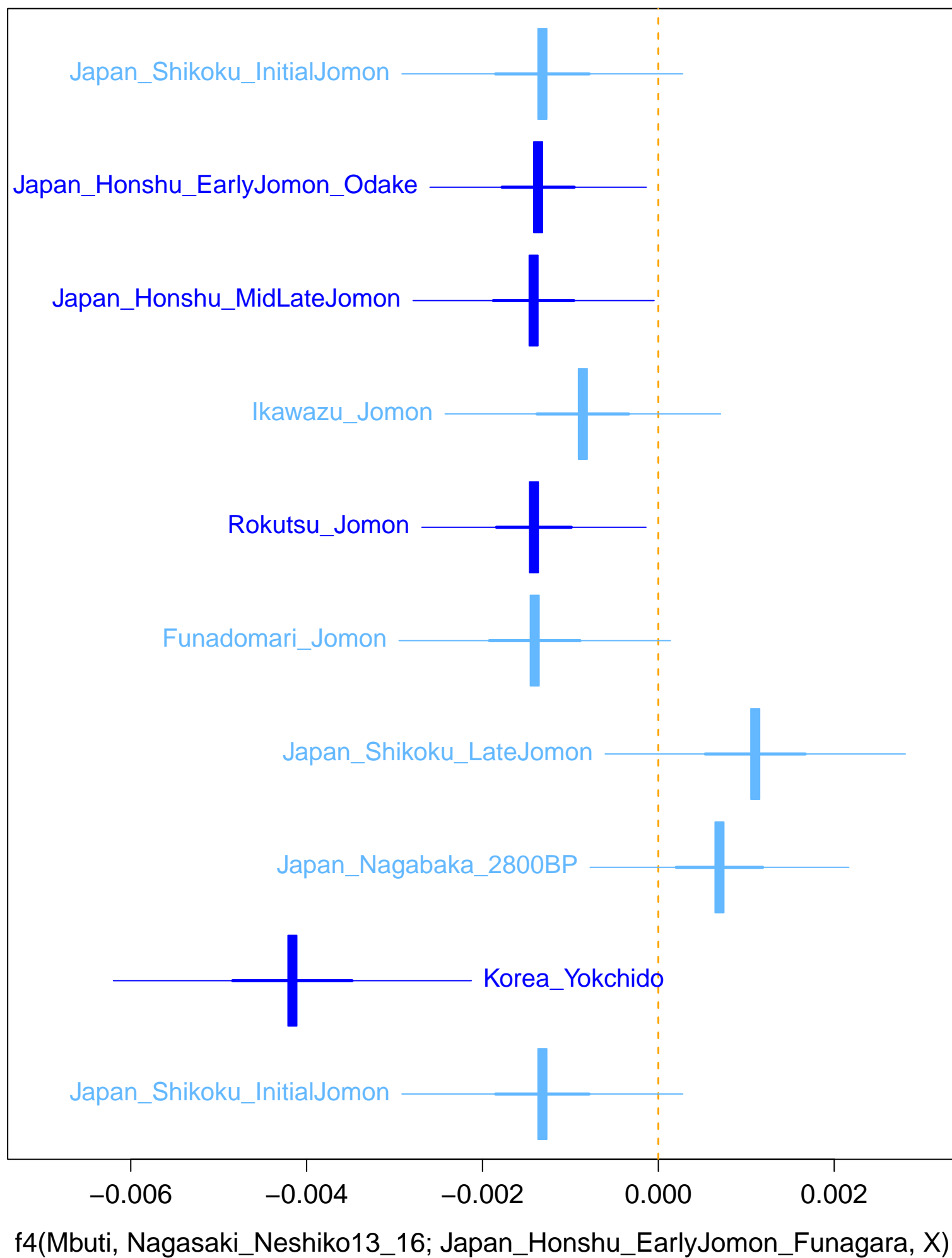

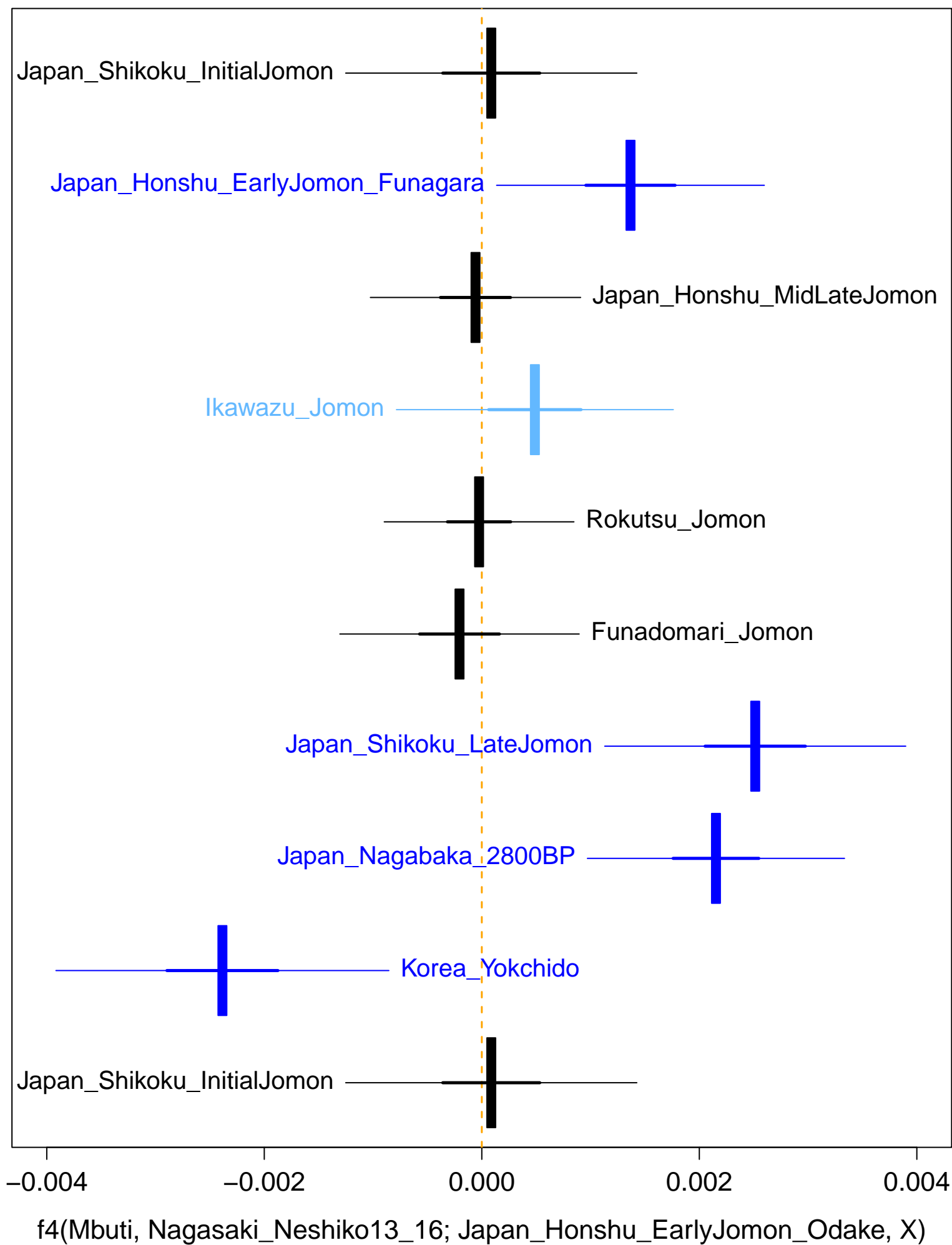

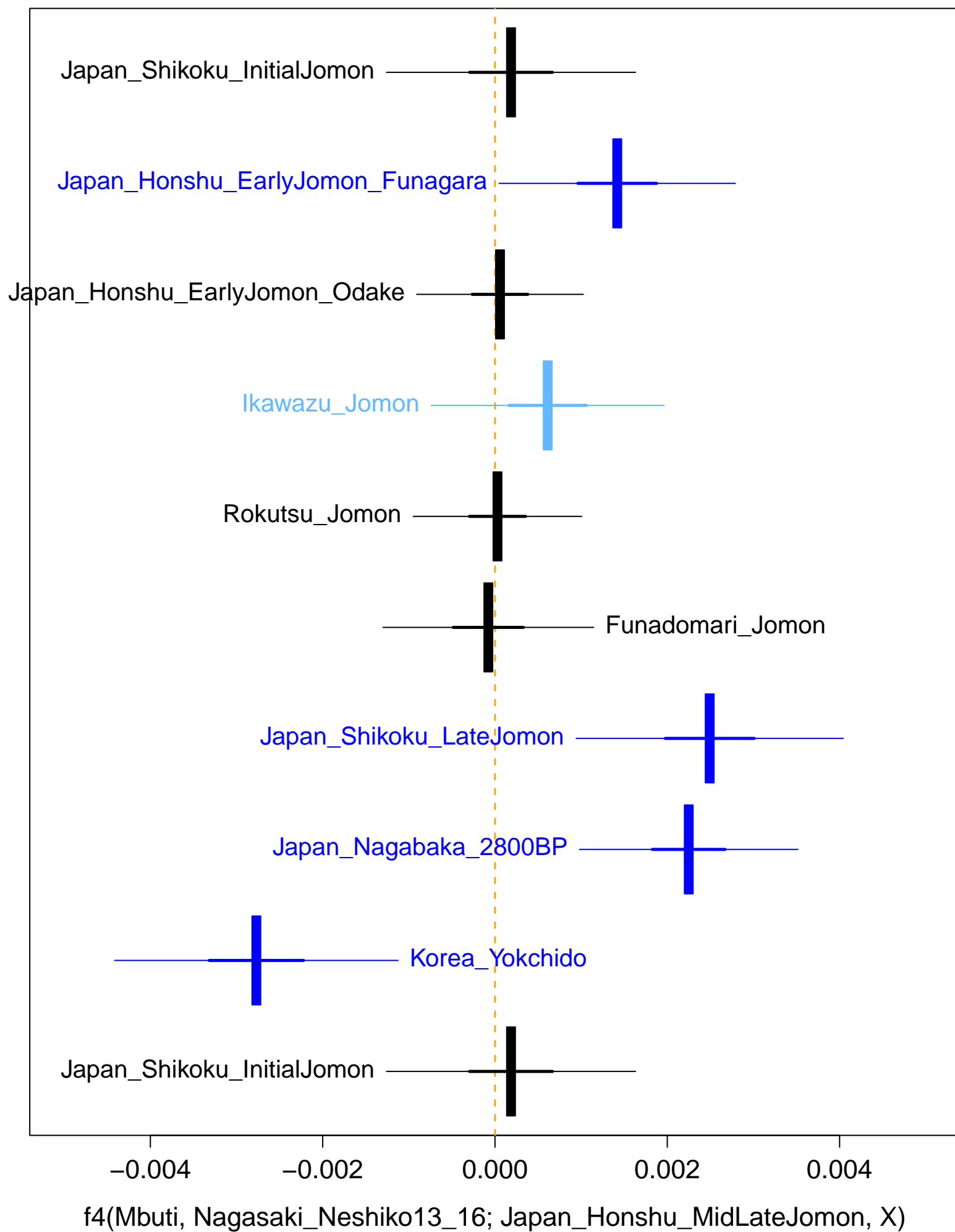

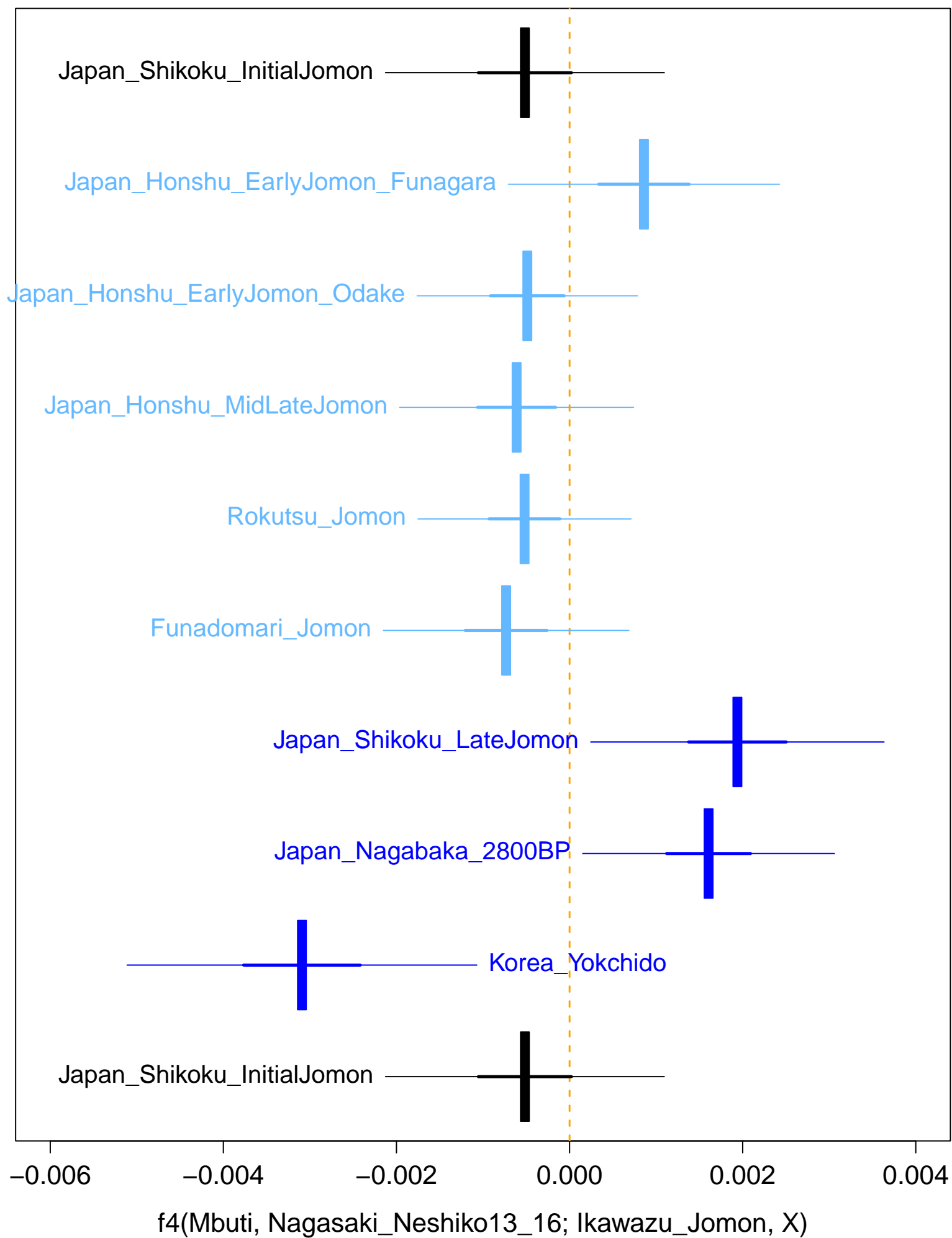

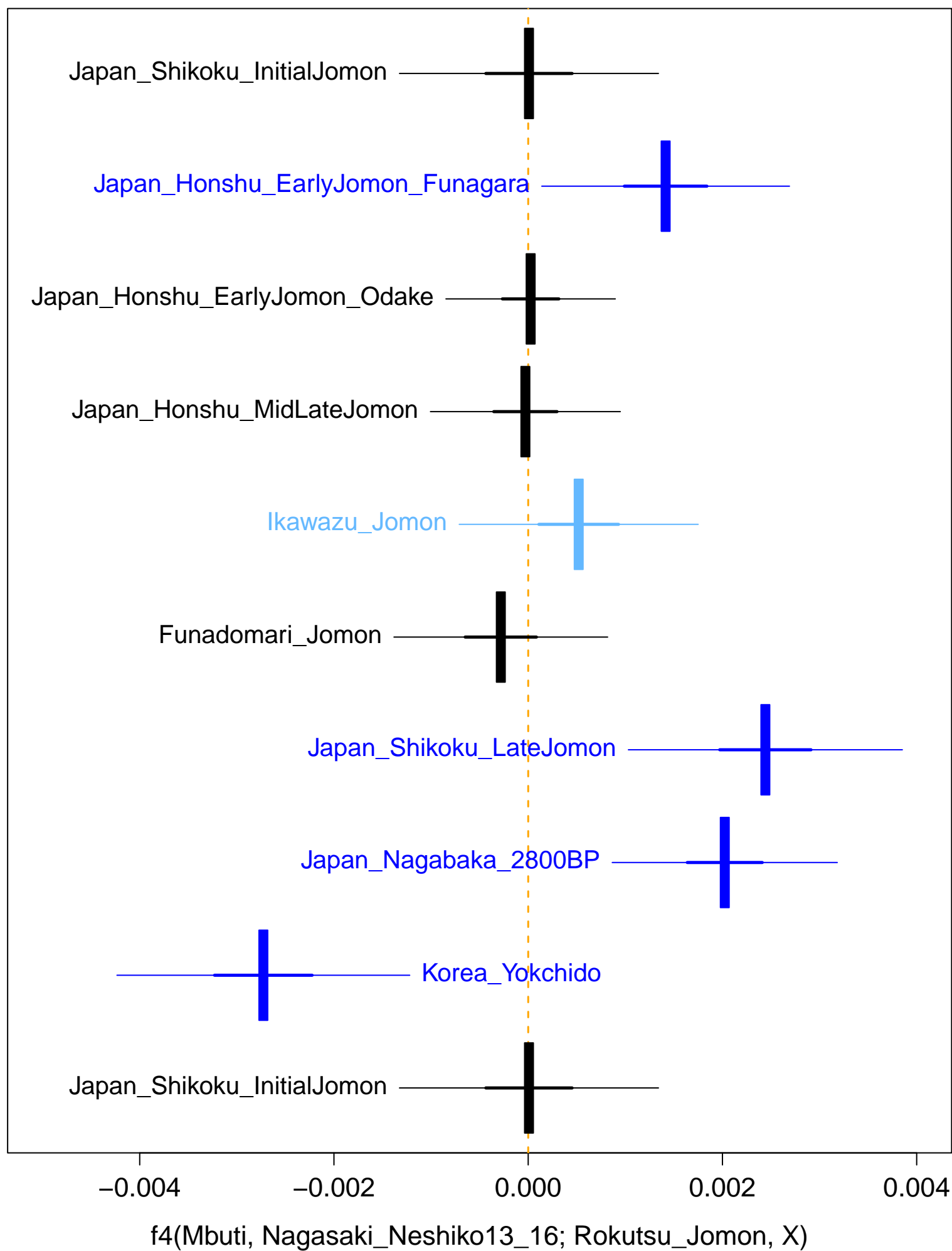

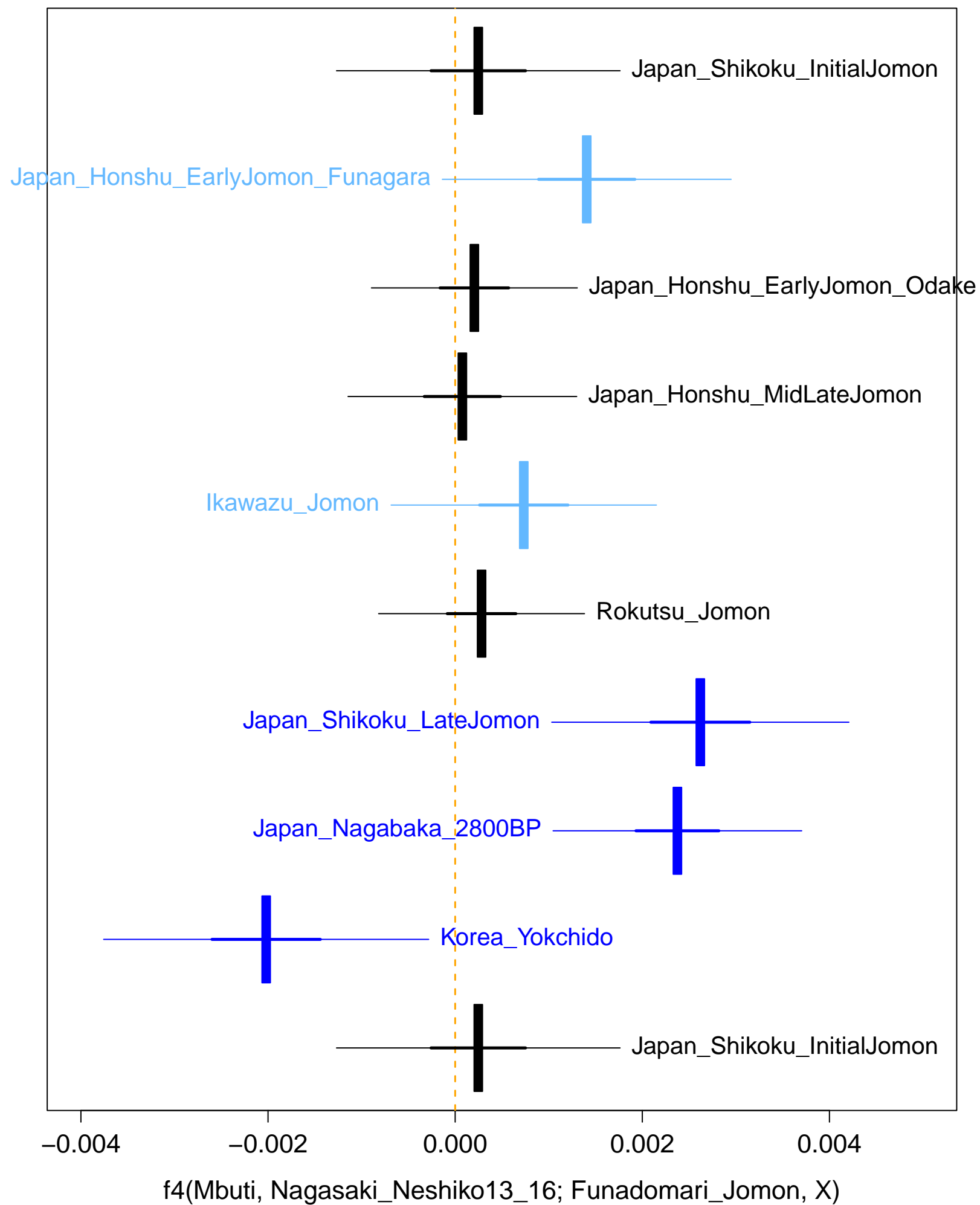

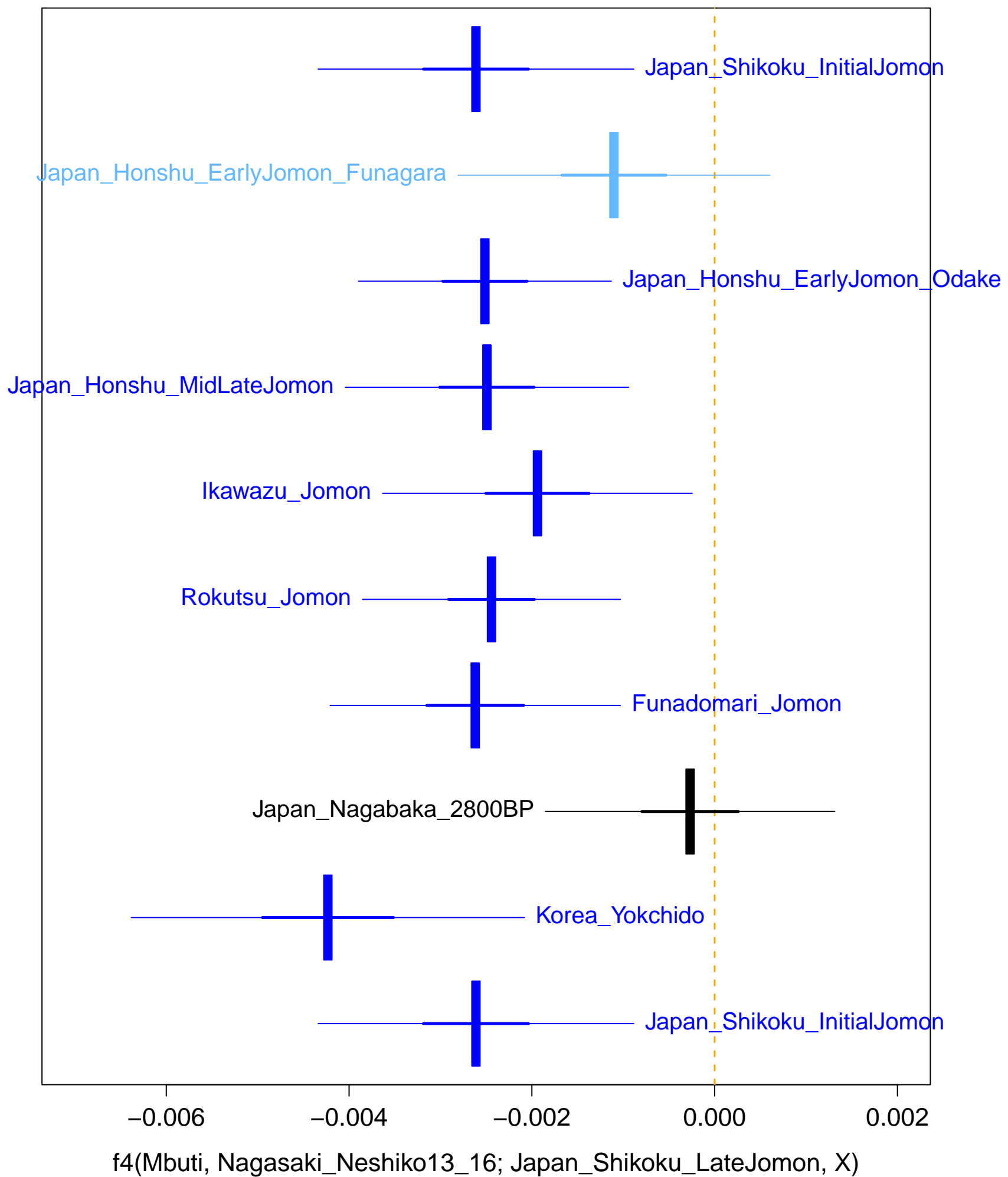

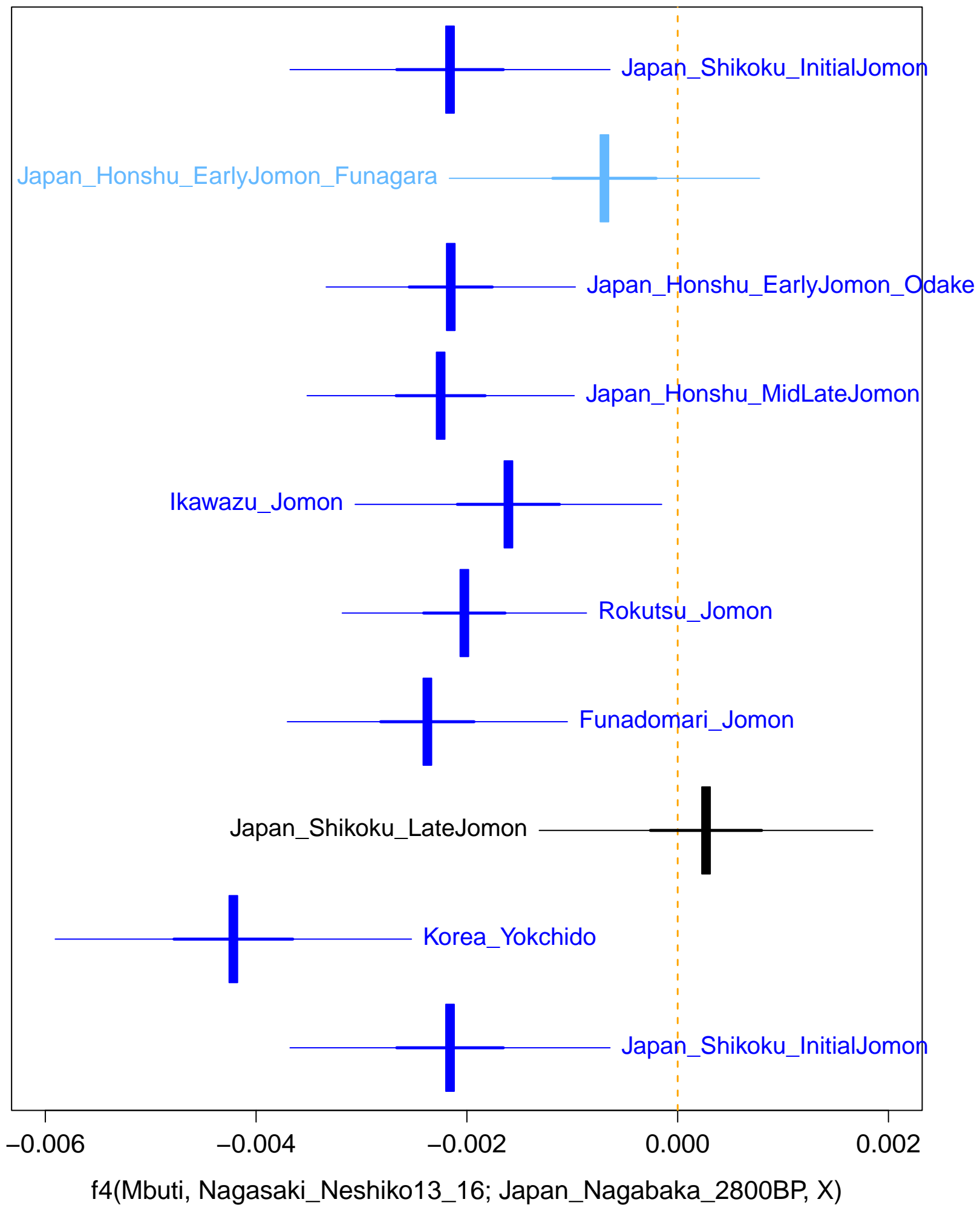

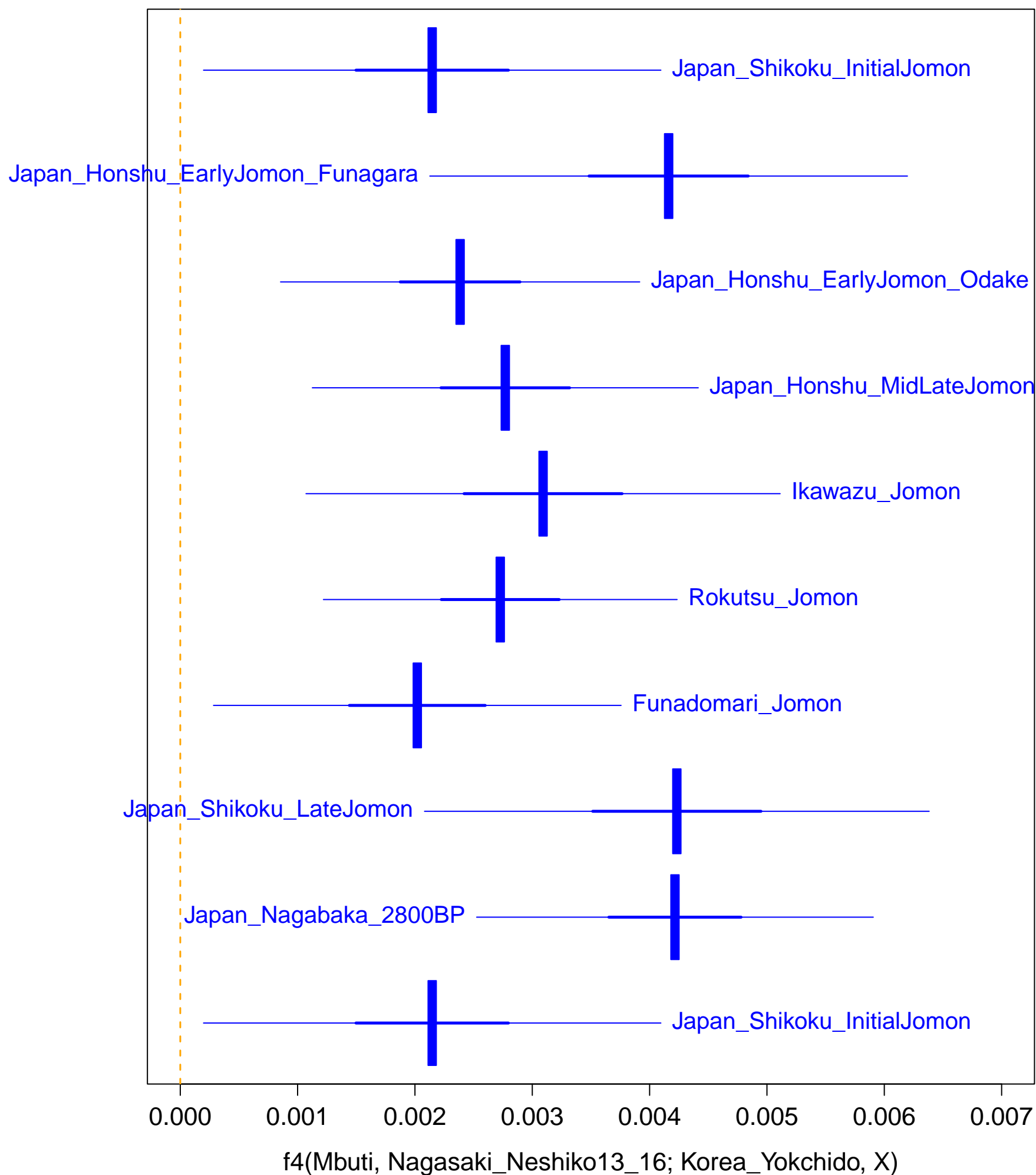

Supplement: Supplementary file 2 — Supplementary Material 2 [file 41598_2026_34996_MOESM2_ESM.pdf]

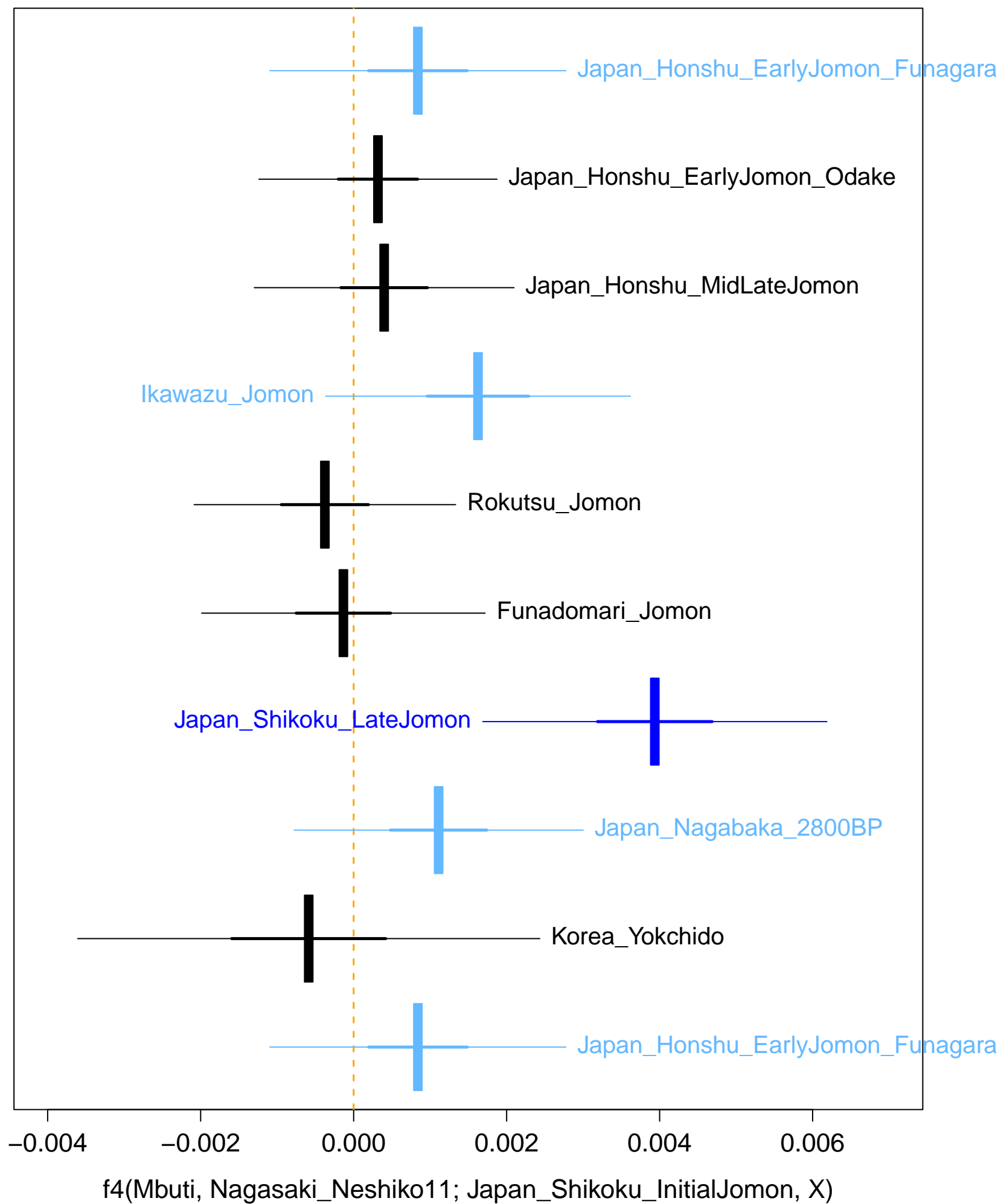

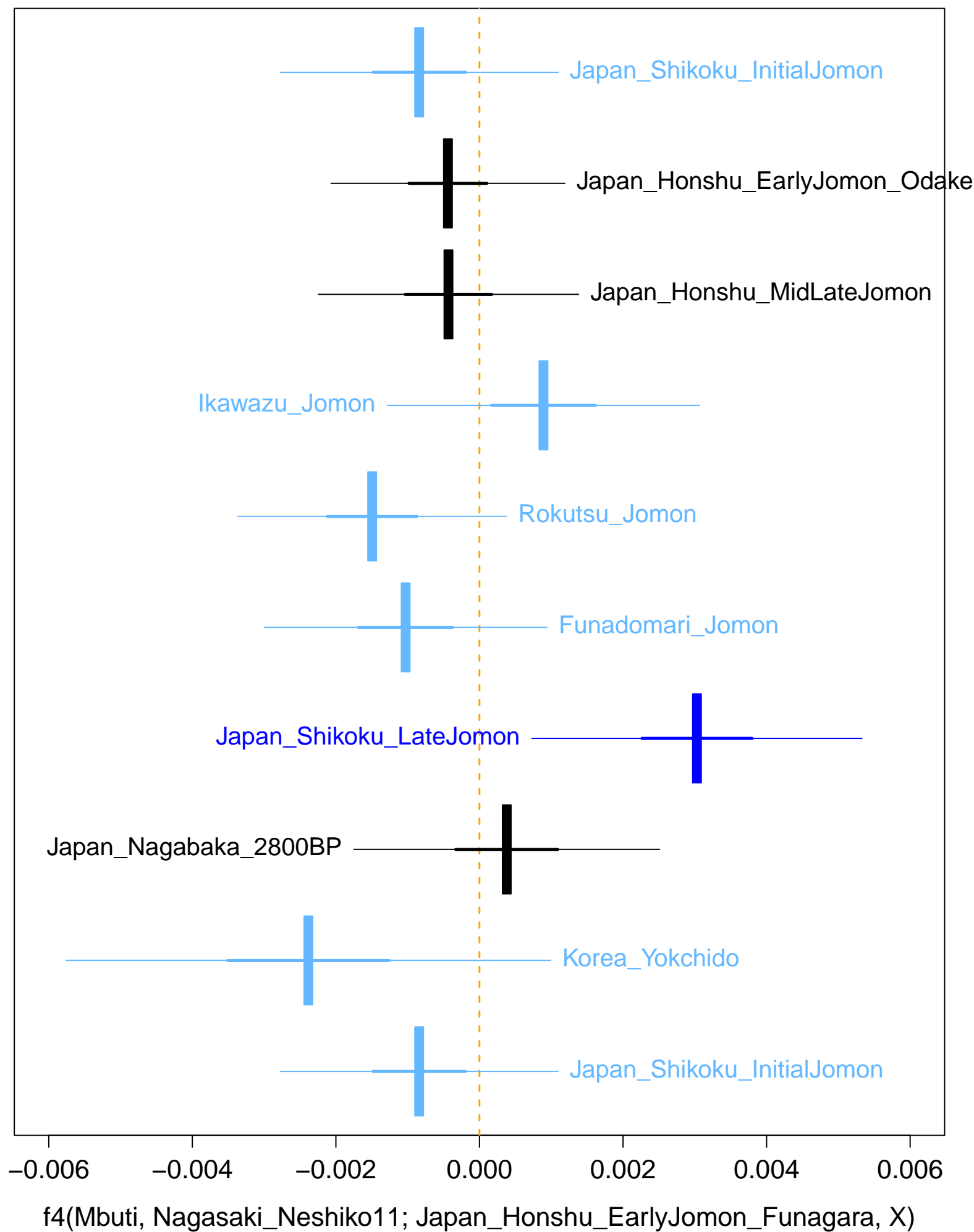

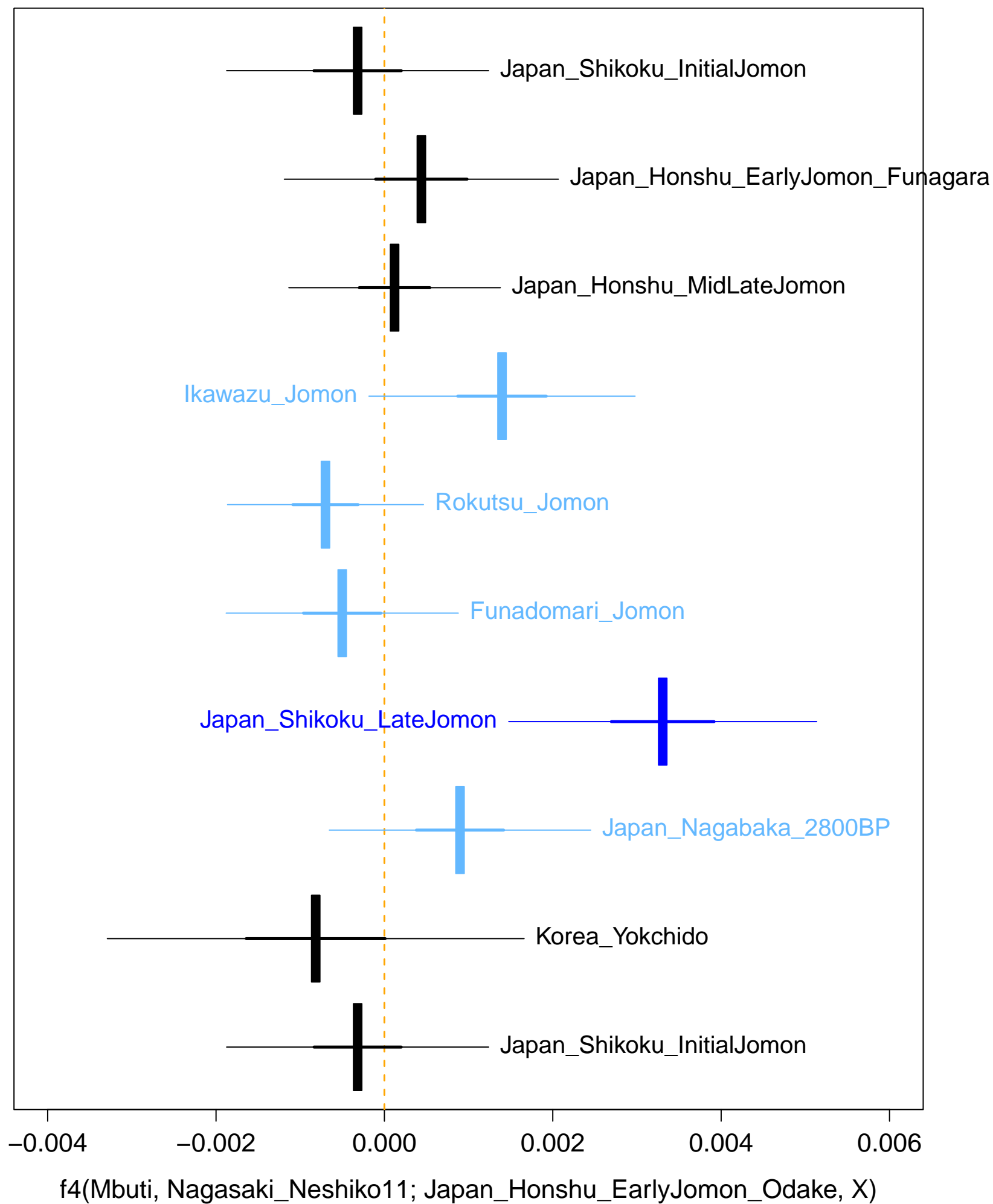

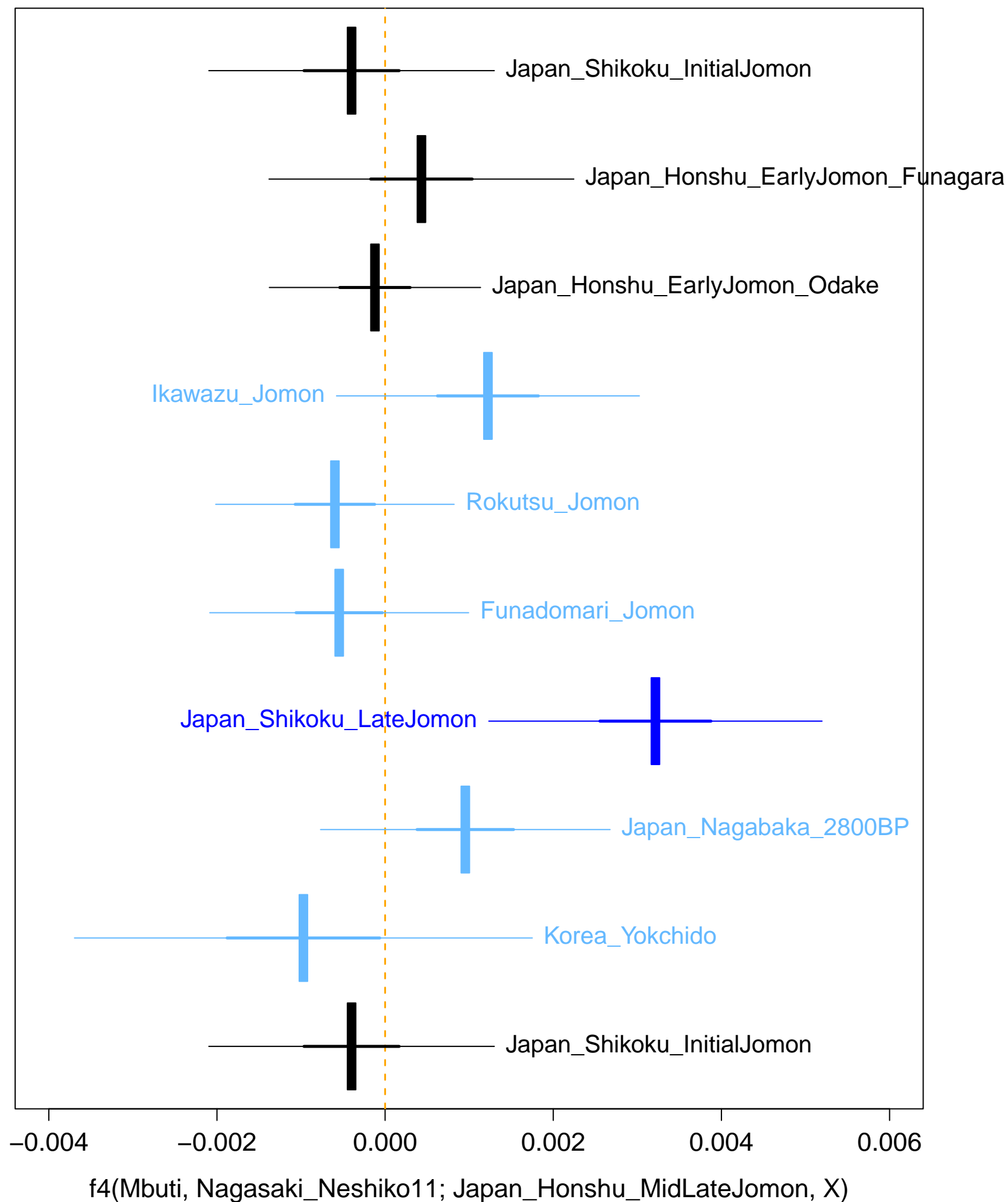

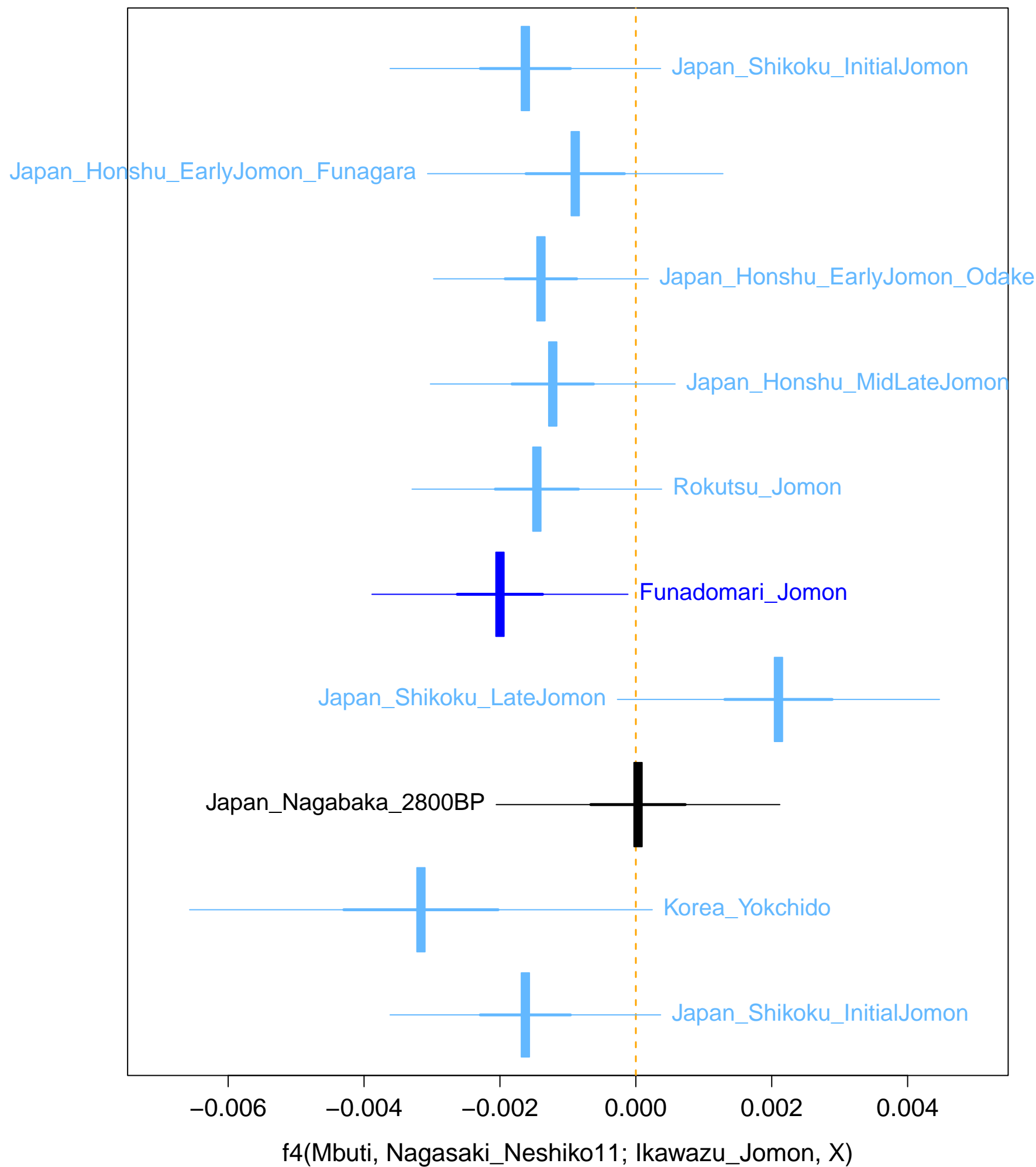

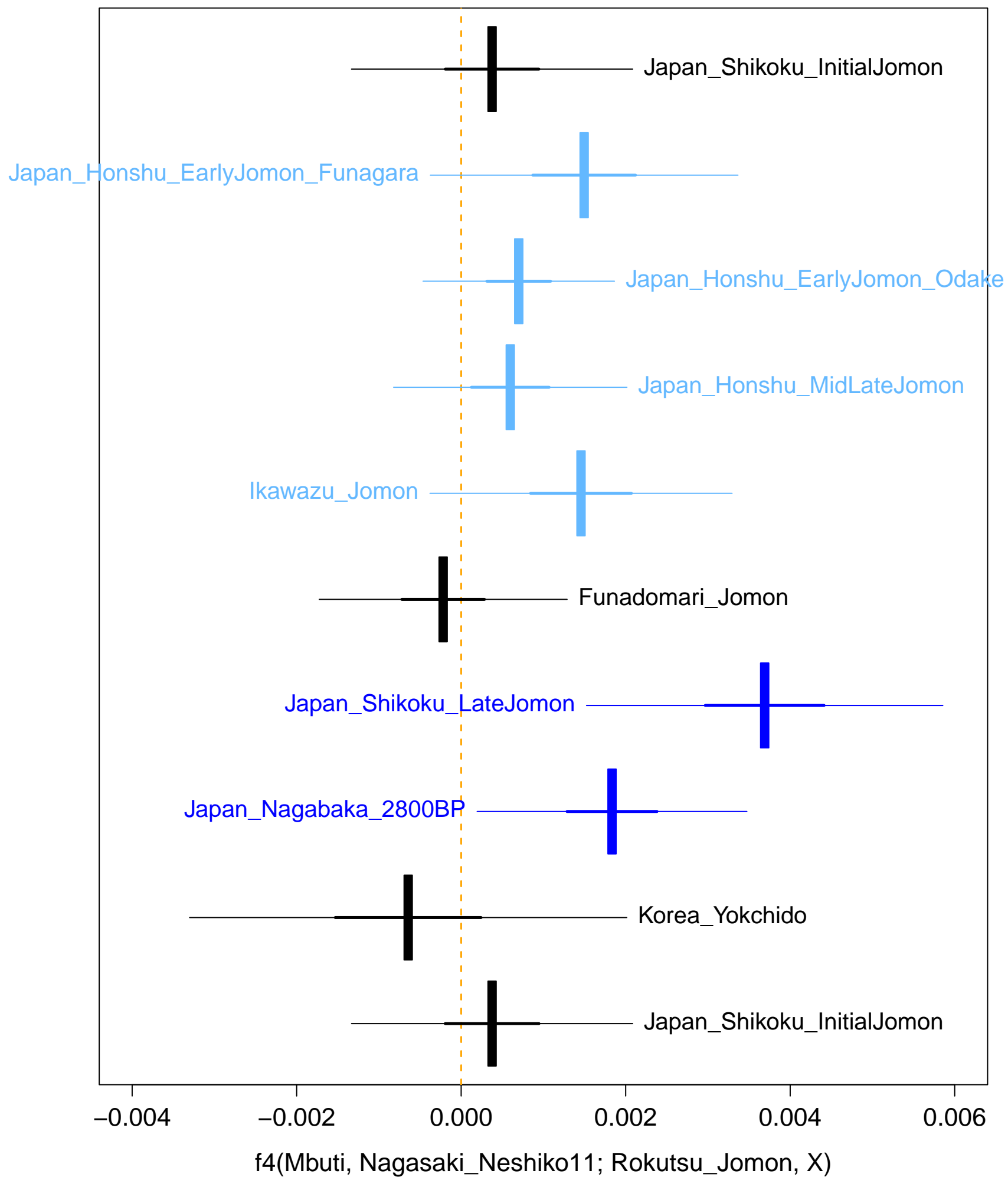

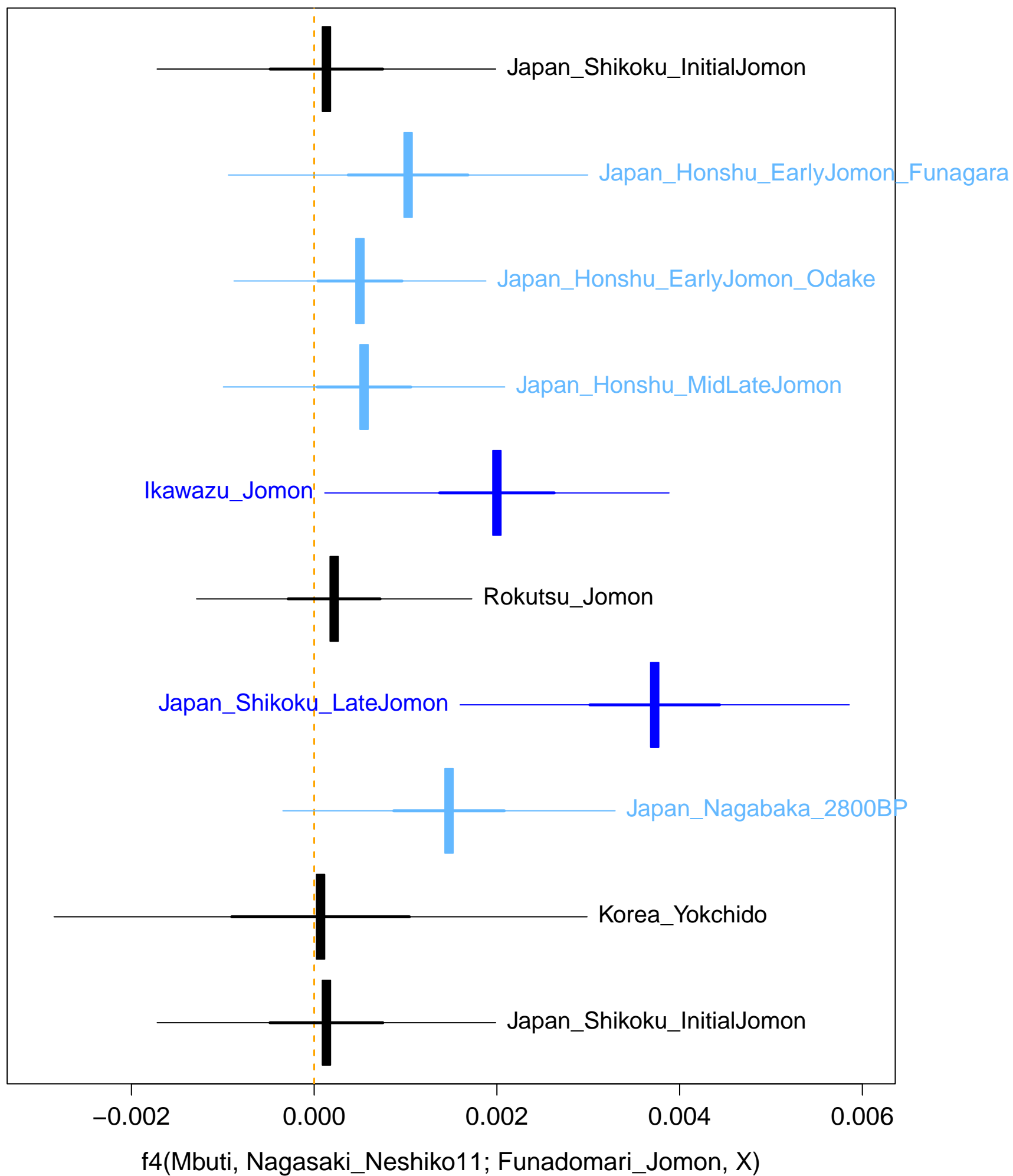

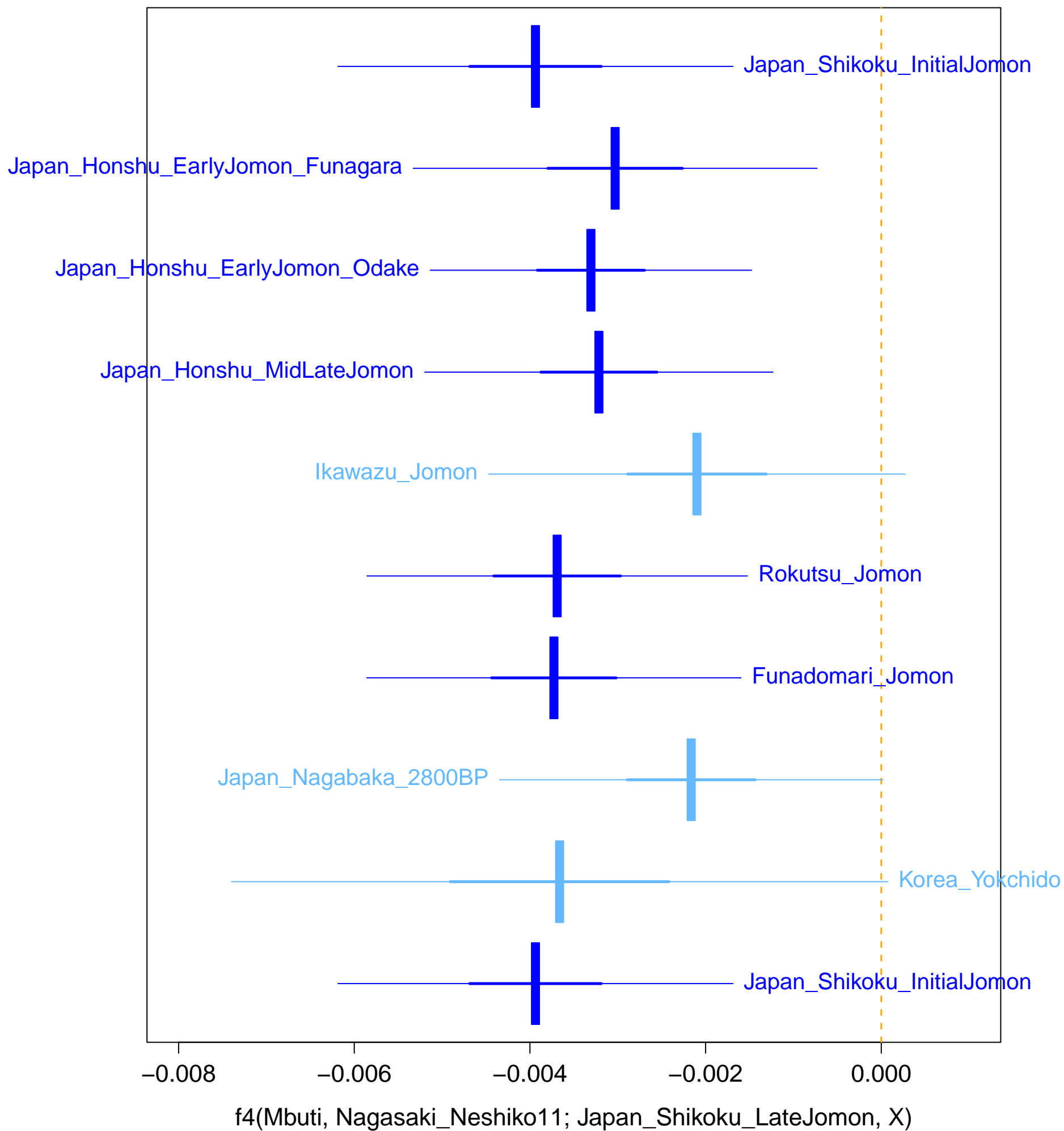

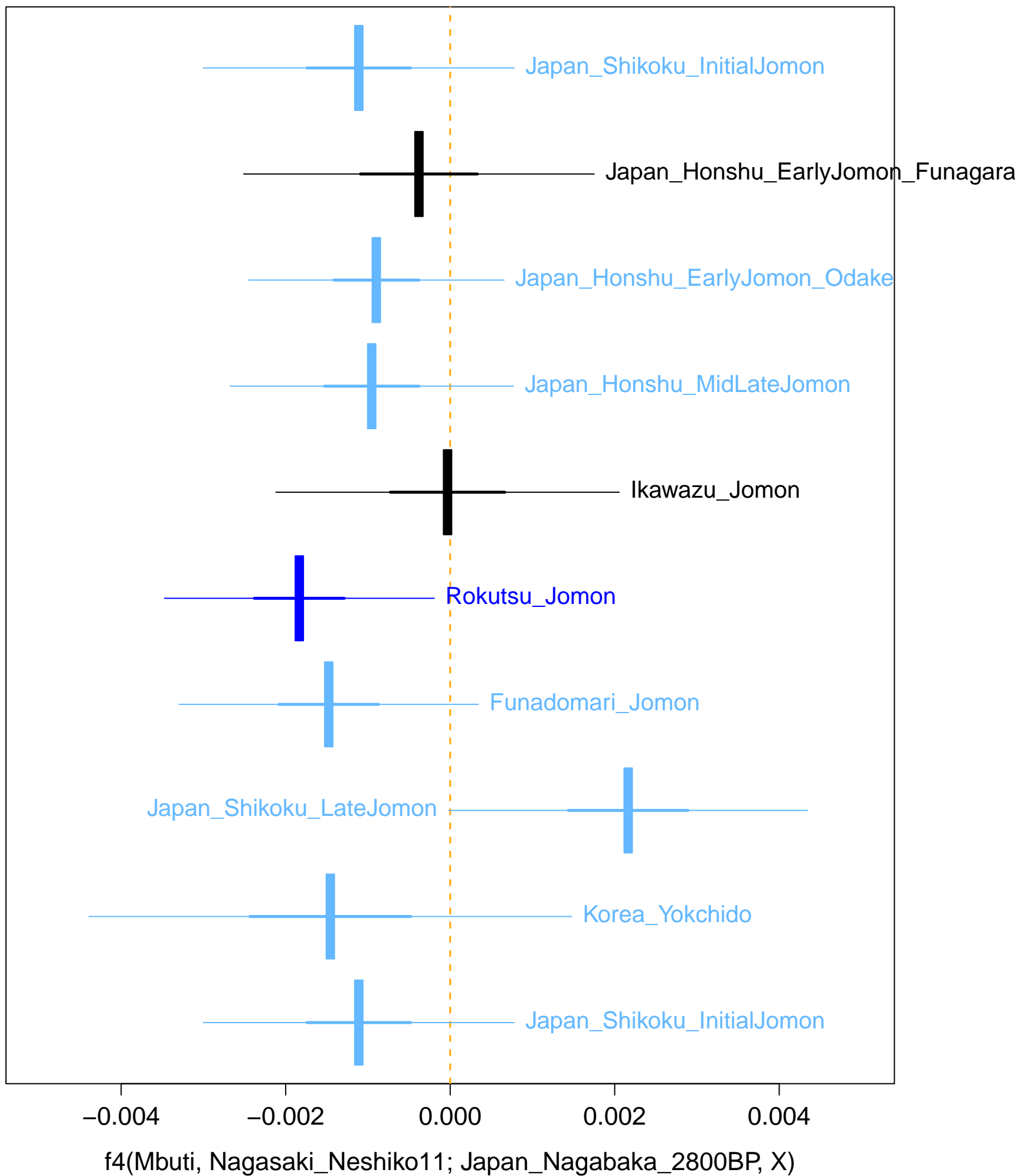

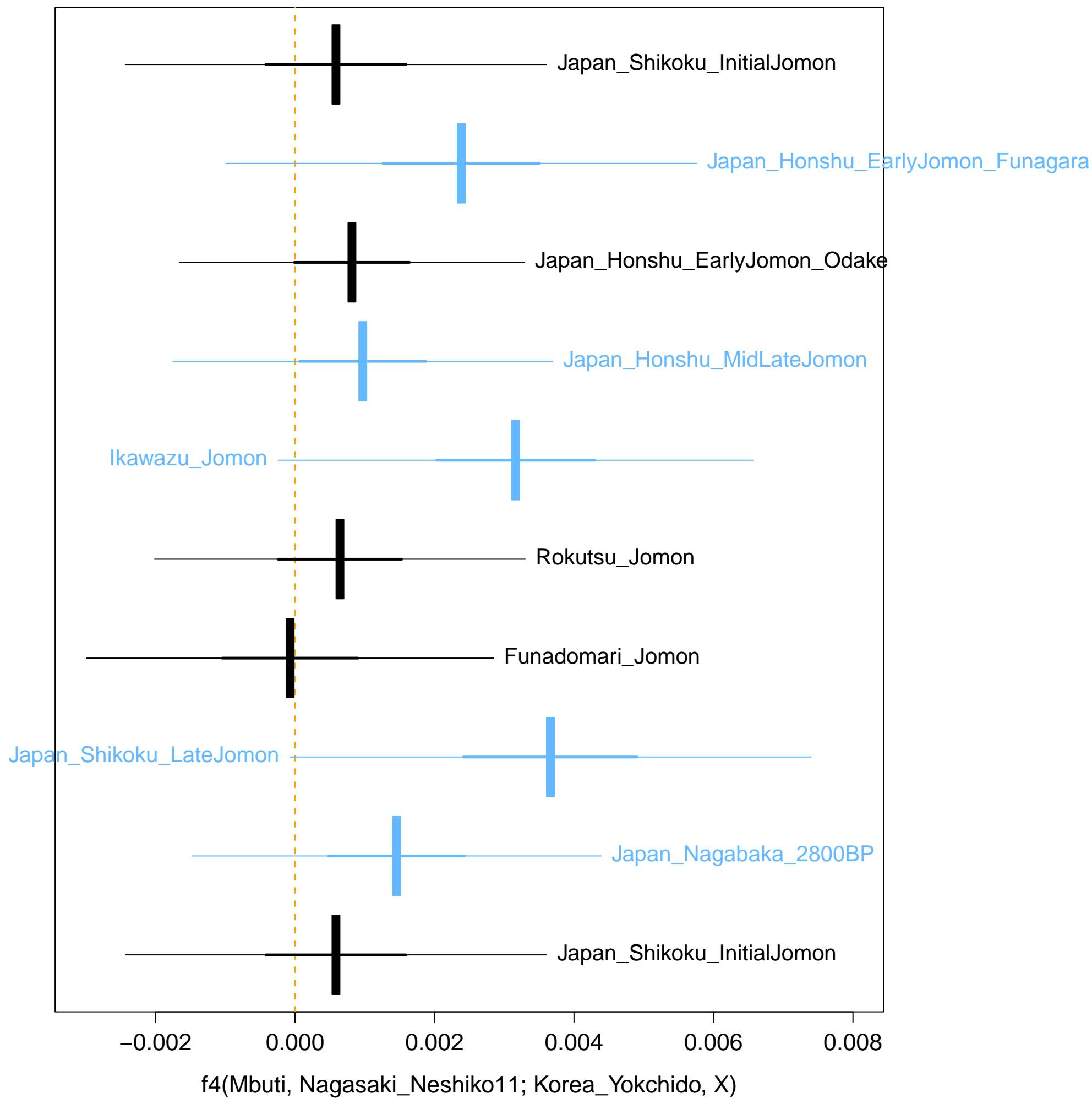

Supplement: Supplementary file 3 — Supplementary Material 3 [file 41598_2026_34996_MOESM3_ESM.pdf]
