# Supplementary material for: Ancient genomes reveal early-stage admixture and genetic diversity in the Northwestern Kyushu Yayoi
Source: Sci Rep. 2026 Jan 7;16:4833. doi: 10.1038/s41598-026-34996-7 (PMC12873284; doi:10.1038/s41598-026-34996-7)
Supplement: Supplementary file 4 — Supplementary Material 4 [file 41598_2026_34996_MOESM4_ESM.pdf]

Supplementary Information

Ancient Genomes Reveal Early-Stage Admixture and Genetic Diversity in the Northwestern Kyushu Yayoi

Jonghyun Kim,[1] Fuzuki Mizuno,[2,*] Takayuki Matsushita,[3] Masami Matsushita,[3] Izumi Naka,[1] Kunihiko Kurosaki,[2] Fuyuki Tokanai,[4] Shintaroh Ueda,[1,2] and Jun Ohashi[1,*]

1. Department of Biological Sciences, Graduate School of Science, The University of Tokyo, Tokyo, 113-0033, Japan.

2. Department of Legal Medicine, Toho University School of Medicine, Tokyo, 143-8540, Japan.

3. The Doigahama Site Anthropological Museum, Yamaguchi, 759-6121, Japan.

4. Center for Accelerator Mass Spectrometry, Yamagata University Advanced Analysis Center, Yamagata, 999-3101, Japan.

*To whom correspondence may be addressed:

Fuzuki Mizuno, Ph.D.

Department of Legal Medicine, Toho University School of Medicine, 5-21-16, Omori-Nishi, Ota-ku, Tokyo, 143-8540, Japan.

Email: fuzuki.mizuno@med.toho-u.ac.jp

Jun Ohashi, Ph.D.

Department of Biological Sciences, Graduate School of Science, The University of Tokyo, 7-3-1, Hongo, Bunkyo-ku, Tokyo, 113-0033, Japan.

Email: jun_ohashi@bs.s.u-tokyo.ac.jp

Table S1. The measured ^{14}C ages, and calibrated ages of the four Middle Yayoi period individuals

Sample name	Annotation in dating analysis	C/N	$\delta^{13}C$ (‰) (IRMS)	$\delta^{15}N$ (‰) (IRMS)	14C age YrBP ± 1σ	Calibrated BC	Median BC
Neshiko11	NSK-11	3.3	-16.32	11.43	2227 ± 20	345BC (7.8%) 281BC<br>258BC (87.6%) 34BC	148
Neshiko13	NSK-13	3.3	-15.30	16.65	2340 ± 23	392BC (95.4%) 149BC	274
Neshiko16	NSK-16	3.4	-15.28	13.91	2355 ± 21	396BC (95.4%) 166BC	286
Shomura	MSMR-No5	3.7	-13.50	10.61	2441 ± 20	506BC (95.4%) 184BC	348

Table S2. Modern DNA contamination estimation

Sample ID	DNA library type	ANGSD (MoM)	verifyBamID
Shomura	Single-stranded library (SS)	0.005546	0.00334688
	Double-stranded library (DS)	0.005252	0.0106953
	merged	0.005801	0.011278
Neshiko11	Single-stranded library (SS)	0.007136	0.0171935
Neshiko13	Single-stranded library (SS)	0.005709	0.0132181
Neshiko16	Single-stranded library (SS)	-	0.019773

Table S3. Mean coverage of shotgun sequenced WGS data of Shomura Yayoi

		**Whole genome**	**X chr**	**Y chr**	**mtDNA**
Nagasaki_ Shomura	mapped reads number	406,705,411	10,841,325	999,516	37,324
	mean coverage	11.5999 x	6.2945 x	1.5648 x	255.28 x

Table S4. Number and percentage of selected non-multiallelic 1240K autosomal SNPs that passed quality filters (DP ≥10, QUAL ≥30, Genotype Quality ≥30)

Sample ID	Number of selected 1240K SNPs	Percentage of called SNPs
Shomura	670,434	58.3 %
Neshiko11	252,331	22.9 %
Neshiko13	1,027,206	89.3 %
Neshiko16	1,069,335	92.9 %

Note: Percentage of called SNPs = (number of successfully selected SNPs) / 1,150,639 (the total number of autosomal SNPs in the 1240K panel). All results presented in this table were obtained from analyses based on diploid genotype data.

Table S5. Quality metrics for 1240K autosomal SNPs obtained using pileupCaller v1.5.2

SampleName	TotalSites	NonMissingCalls	avgRawReads	avgDamageCleanedReads	avgSampledFrom
Shomura	1150639	1144670	12.116394455602496	12.116394455602496	12.106734605727773
Neshiko11	1150639	1137198	7.764730727882507	7.764730727882507	7.752436689526428
Neshiko13	1150639	1147173	24.67114881383301	24.67114881383301	24.645463955245738
Neshiko16	1150639	1148642	40.681202357994124	40.681202357994124	40.61968697393361

Note: Quality metrics were generated using pileupCaller v1.5.2. Definitions for each column are as follows:

– SampleName: Sample identifier.
– TotalSites: Total number of targeted 1240K autosomal SNP sites (before transition filtering).
– NonMissingCalls: Number of SNP sites with non-missing pseudo-haploid calls (before transition filtering).
– avgRawReads: Mean coverage of raw pileup input data across all targeted sites.
– avgDamageCleanedReads: Mean coverage after removal of single-stranded damage.
– avgSampledFrom: Mean coverage after excluding reads containing tri-allelic positions.

Table S6. Sex determination

	**X chr reads**	**Y chr reads**	**Ry index**	**95% CI**	**Genetic sex**
Shomura	10,891,084	1,007,053	0.0846	1.28×10^{-8}	Male
Neshiko11	847,773	313,782	0.270	3.33×10^{-7}	Male
Neshiko13	2,051,227	887,648	0.302	1.41×10^{-7}	Male
Neshiko16	6,346,965	9,882	0.00155	4.79×10^{-10}	Female

Table S7. TRUFFLE-based estimates of IBD0, IBD1, and IBD2 probabilities

**Individual 1**	**Individual 2**	**IBD0**	**IBD1**	**IBD2**
Shomura	Neshiko11	1.000000	0.000000	0.000000
Shomura	Neshiko13	0.998309	0.000000	0.001691
Shomura	Neshiko16	0.998884	0.000000	0.001116
Neshiko11	Neshiko13	0.993617	0.005302	0.001081
Neshiko11	Neshiko16	0.997044	0.000000	0.002956
Neshiko13	Neshiko16	0.715681	0.264951	0.019368

Table S8. Results of qpAdm admixture modeling

**Mainland Jomon as Source (1: mainland Jomon, 2: modern Korean (SGDP))**						
**Target**	***P*-value**	**c1**	**c2**	**SE1**	**SE2**	**# SNPs**
Nagasaki_Shomura	0.0846671205	0.311	0.689	0.039	0.039	440,796
Nagasaki_Neshiko11	0.281349175	0.515	0.485	0.048	0.048	177,677
Shimomotoyama_Yayoi	0.676721811	0.768	0.232	0.106	0.106	79,689
Doigahama_Yayoi	0.833649667	0.117	0.883	0.041	0.041	596,672
Japan_KumaNishioda_Yayoi	0.168053487	0.155	0.845	0.118	0.118	73,887
Japan_Honshu_Kofun	0.188273415	0.113	0.887	0.028	0.028	690,247
Japan_Nagabaka_historic	0.179433657	0.318	0.682	0.028	0.028	640,362
Japanese	0.117894999	0.087	0.913	0.021	0.021	626,634
**(B) Neshiko 13/16 as Source (1: Neshiko 13/16, 2: modern Korean (SGDP))**						
**Target**	***P*-value**	**c1**	**c2**	**SE1**	**SE2**	**# SNPs**
Nagasaki_Shomura	0.295973063	0.351	0.649	0.043	0.043	456,398
Nagasaki_Neshiko11	0.625753702	0.57	0.43	0.053	0.053	186,727
Shimomotoyama_Yayoi	0.935257497	0.94	0.06	0.136	0.136	79,753
Doigahama_Yayoi	0.892821355	0.13	0.87	0.045	0.045	613,769
Japan_KumaNishioda_Yayoi	0.187794033	0.242	0.758	0.144	0.144	73,837
Japan_Honshu_Kofun	0.206496616	0.131	0.869	0.031	0.031	695,275
Japan_Nagabaka_historic	0.404951651	0.354	0.646	0.032	0.032	643,361
Japanese	0.190177198	0.104	0.896	0.023	0.023	612,418

The notation c_i (for $i = 1, 2$) denotes the admixture proportion derived from the i-th ancestry, and SE_i represents the standard error associated with c_i.

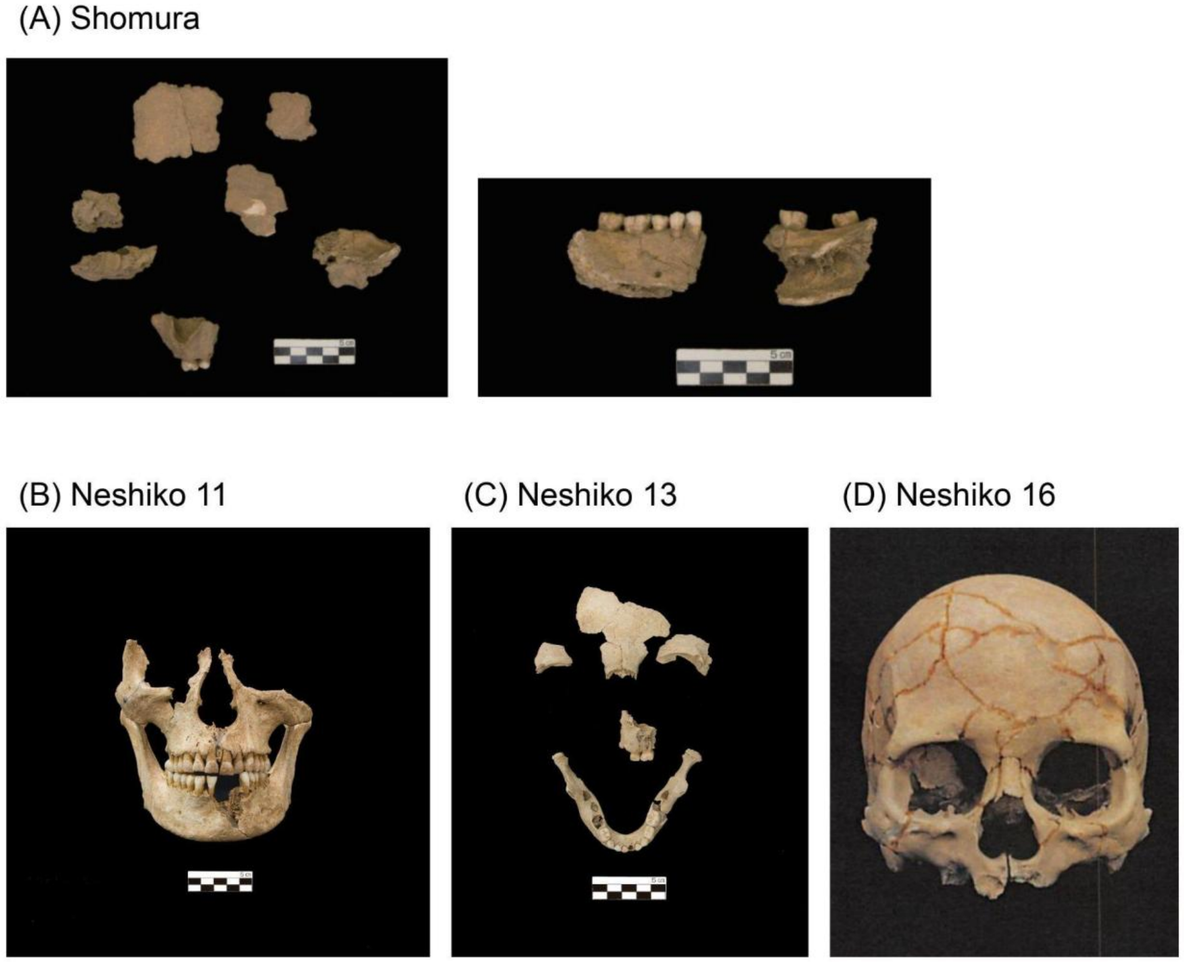

Figure S1. Skull remains of newly analyzed ancient individuals

Skull photos of each individual taken at the Doigahama Anthropological Museum. (A) The Yayoi individuals from the Shomura site had incomplete remaining bones, making it difficult to determine whether he exhibited Jomon or Yayoi characteristics morphologically. (B)~(D) The Yayoi period individuals from the Neshiko site exhibited morphological characteristics similar to those of the Jomon people, such as a low, wide face and short stature.

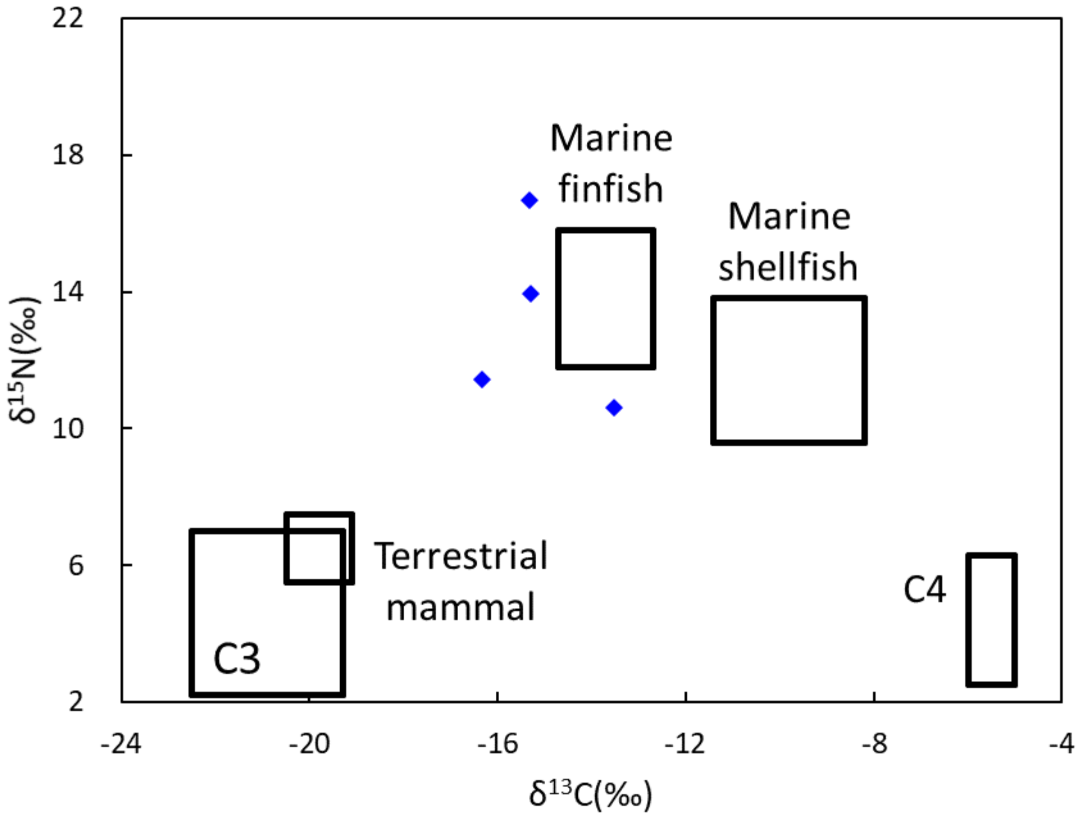

Figure S2. Stable isotope values of human bone collagen and dietary references.
$\delta^{13}C$ and $\delta^{15}N$ values of human bone collagen from the analyzed samples, shown in comparison with estimated isotopic ranges of various food resources (after Yoneda et al., 2004).

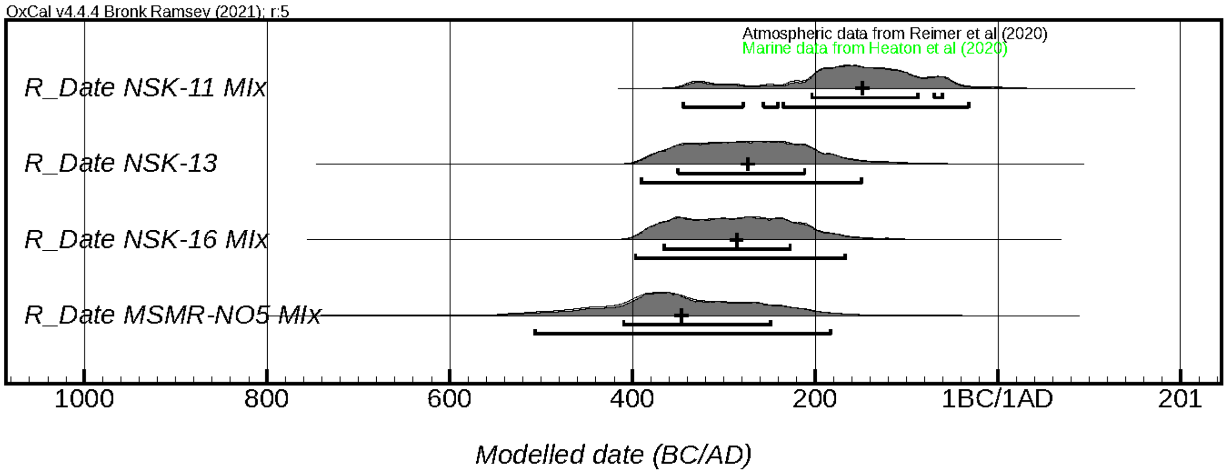

Figure S3. Probability distributions of the calibrated radiocarbon dates for the four collagen samples.

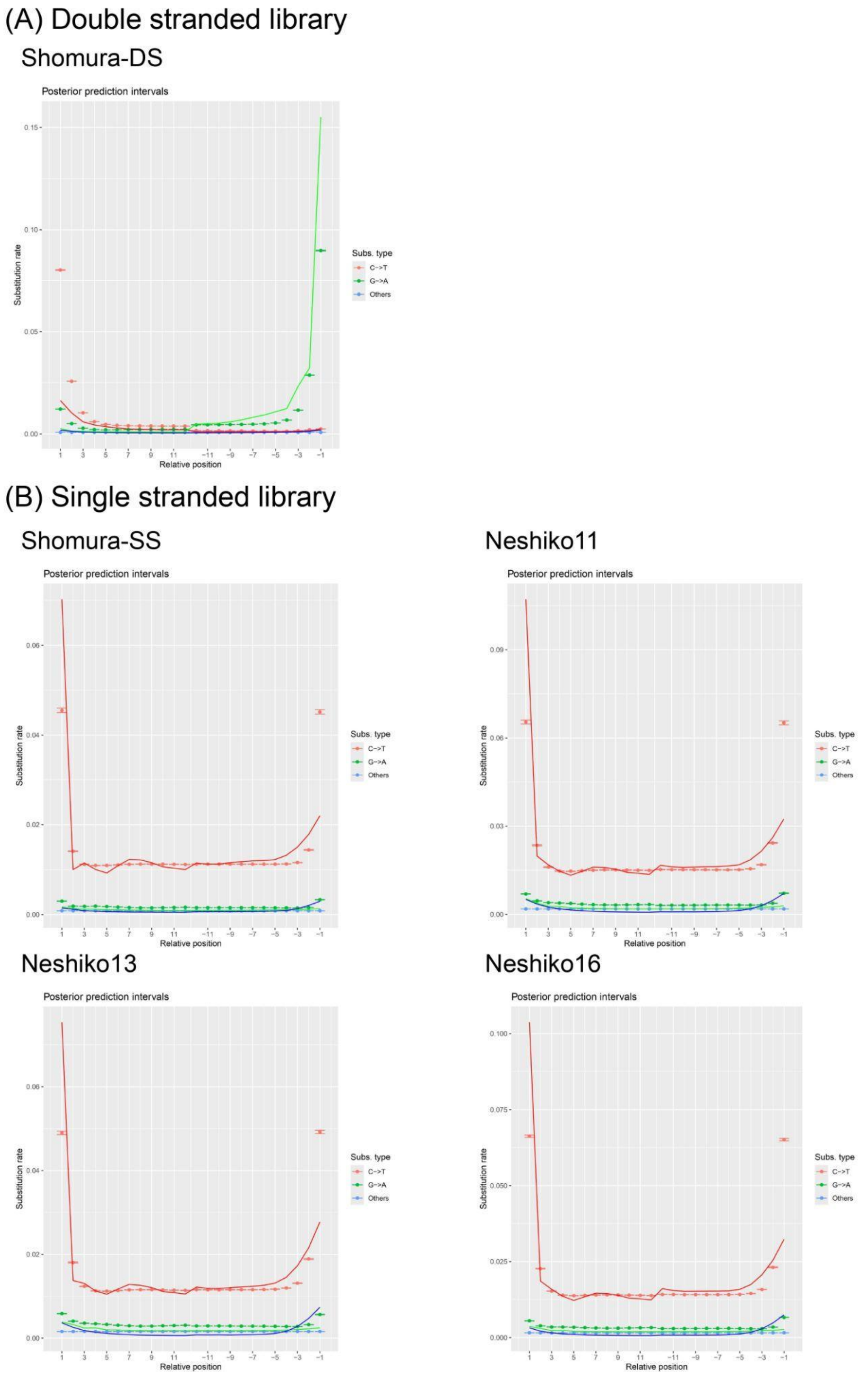

Figure S4. Deamination patterns in ancient DNA libraries.

Empirical misincorporation frequency plots with posterior predictive intervals from the fitted model for each library, generated using mapDamage v2.2.2. (A) Double-stranded DNA library showing a high frequency of C-to-T transitions at the 5′ end and G-to-A transitions at the 3′ end. (B) Single-stranded DNA library showing a high frequency of C-to-T transitions at both ends of the reads.

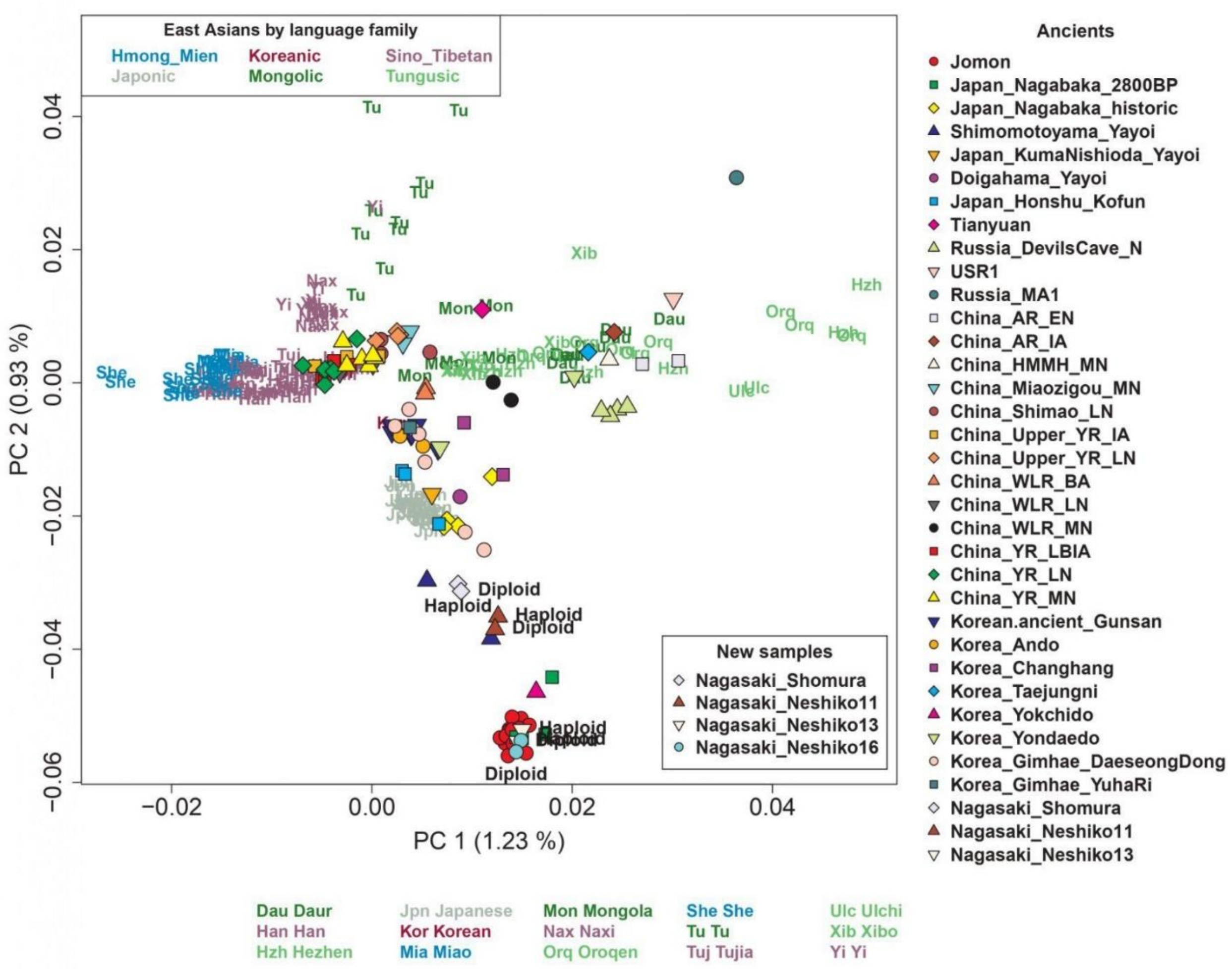

Figure S5. PCA plot including pseudo-haploid genotype data of the target samples.

Pseudo-haploid and diploid genotype data from the same individuals are indicated by the same shape. All other individuals included in the PCA are identical to those shown in Figure 2.

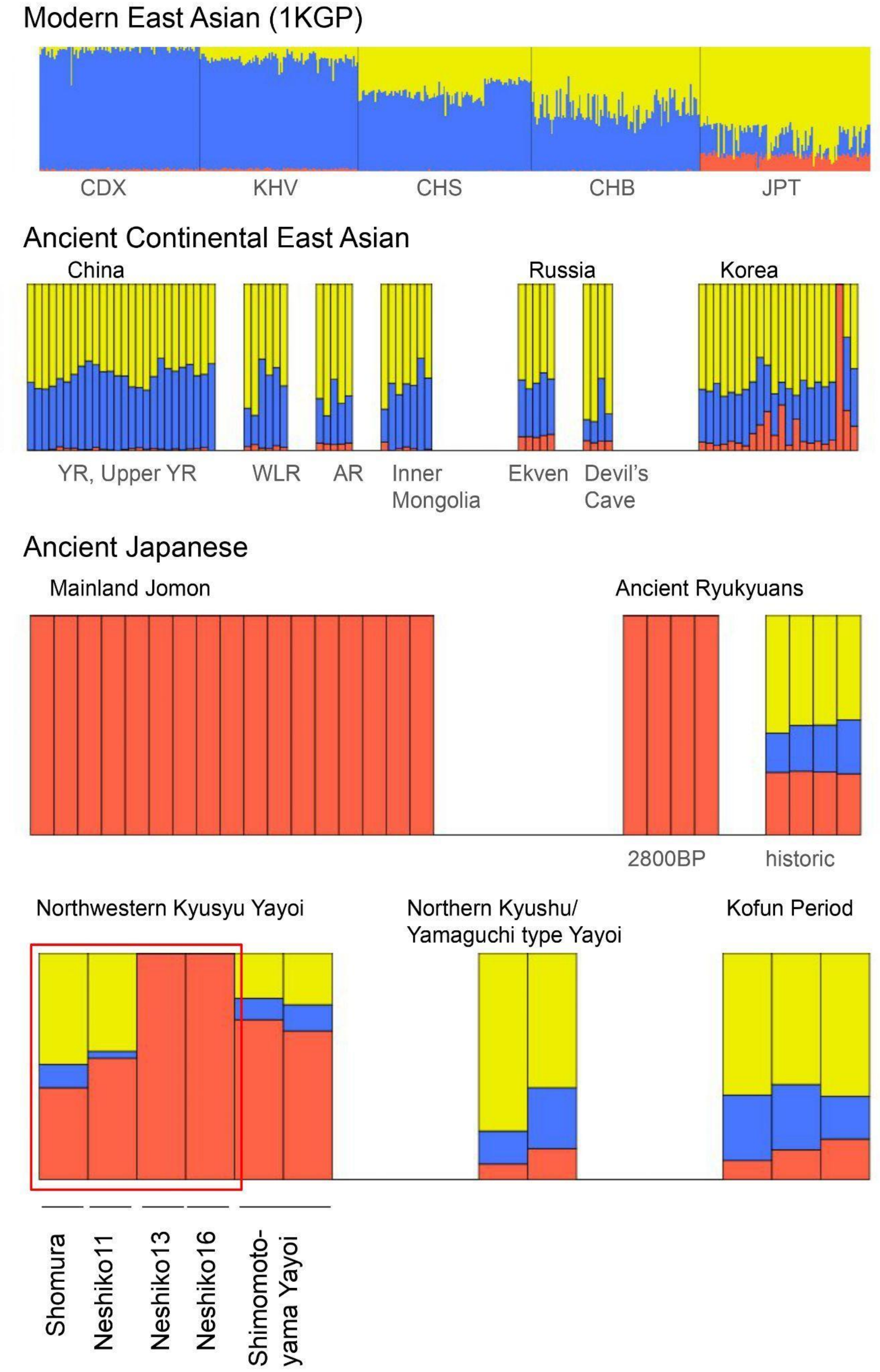

Figure S6. ADMIXTURE analysis.

Bar plots showing estimated ancestry proportions for East Asian and Northeast Asian individuals at *K*=3. In this analysis, pseudo-haploid genotypes were used for the four Northwestern Kyushu Yayoi individuals.

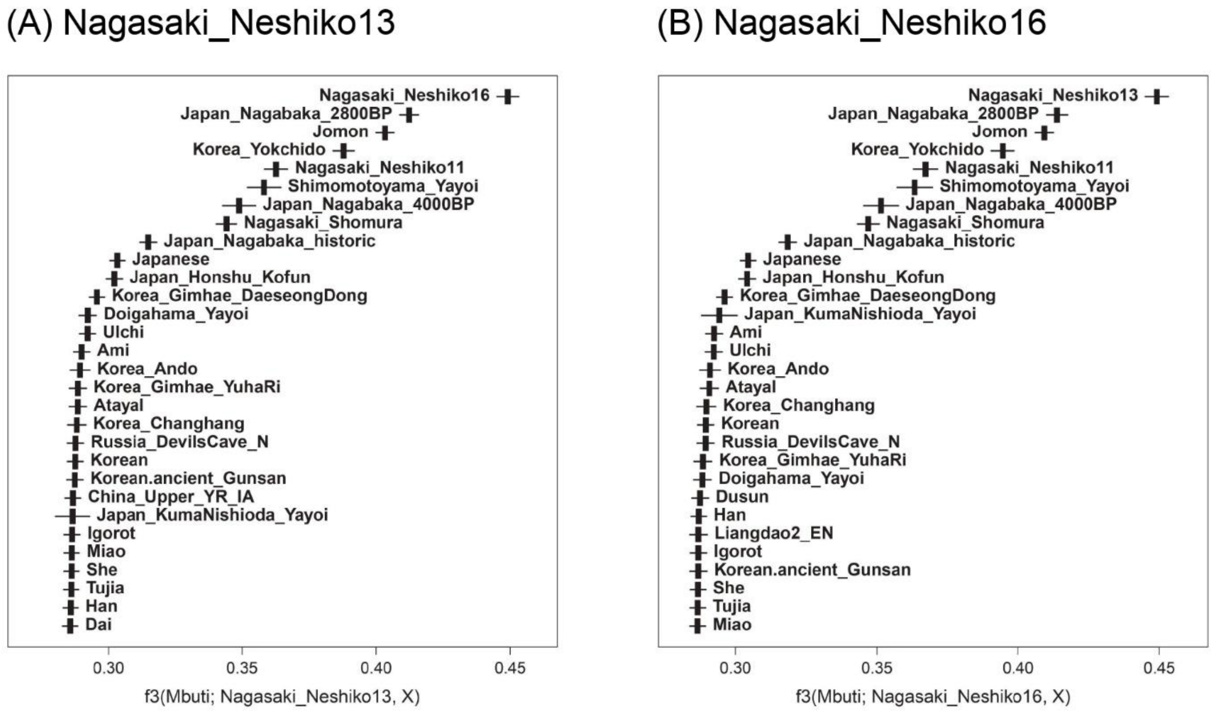

Figure S7. Outgroup *f*3-statistics for Neshiko13 and Neshiko16.

Outgroup *f*3 statistics of the form *f*3(Mbuti; target, X) were calculated using (A) Neshiko13 and (B) Neshiko16 as the target individual in each analysis. The top 30 populations with the highest *f*3 values for each target in our dataset are shown (i.e., the X populations displayed differ between the two targets). Error bars represent the range of $|Z| \leq 1$.

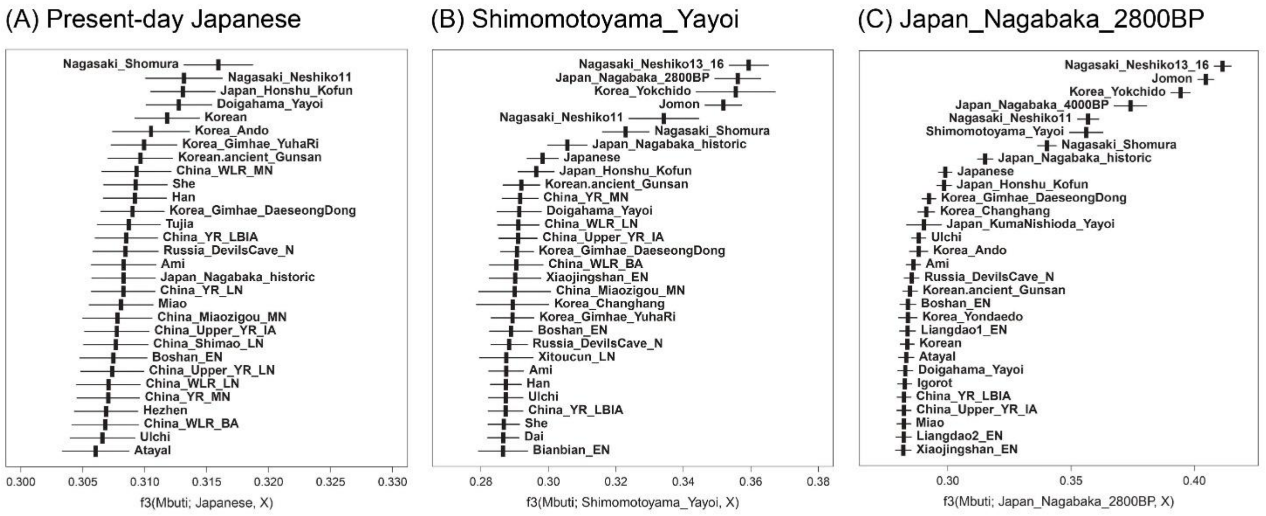

Figure S8. Outgroup *f*3-statistics for present-day Japanese, Shimomotoyama_Yayoi and Japan_Nagabaka_2800BP.

Outgroup *f*3-statistics of the form *f*3(Mbuti; target, X) were calculated using the following populations as targets: (A) present-day Japanese; (B) Shimomotoyama Yayoi, reported to belong to the Northwestern Kyushu Yayoi; and (C) Japan_Nagabaka_2800BP from the Nagabaka site in the Ryukyu archipelago, which has been reported to be genetically identical to the mainland Jomon. The top 30 populations with the highest *f*3 values for each target in our dataset are shown (i.e., the X populations displayed differ for each target). Error bars represent the range of $|Z| \leq 1$.

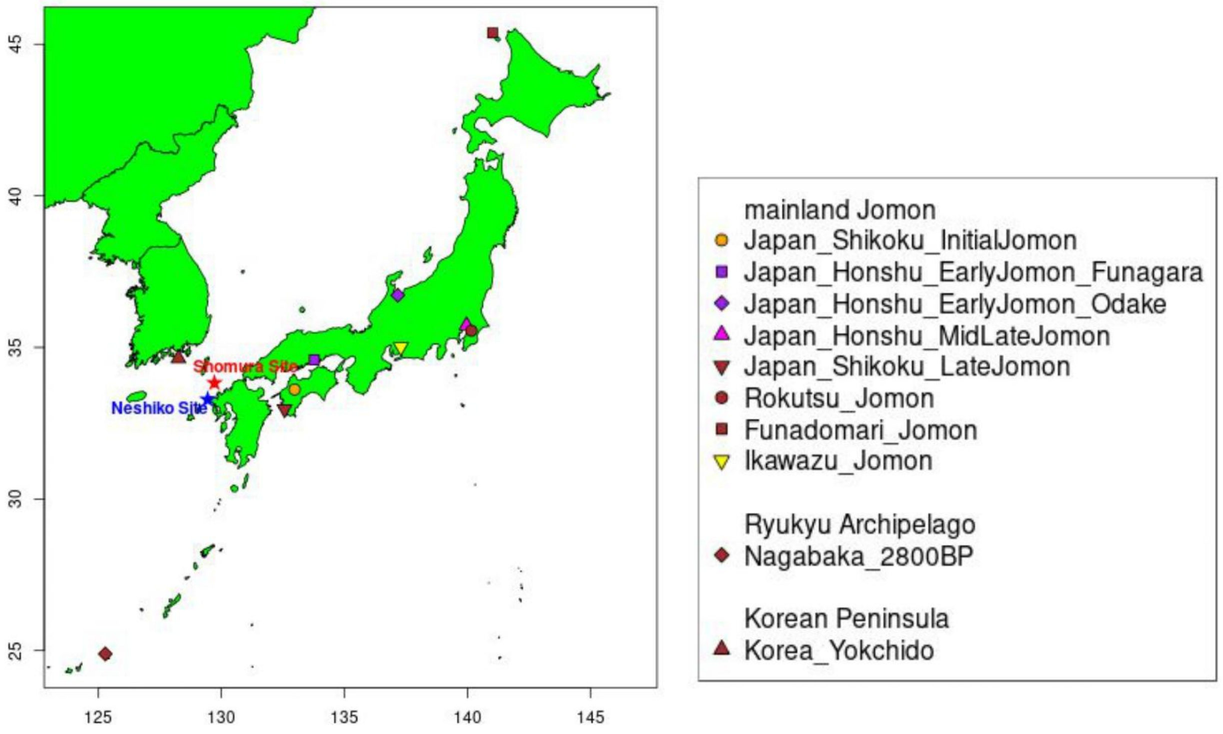

Figure S9. Geographical and temporal information for each Jomon population

This map shows the archaeological sites from which the Jomon individuals included in our analyses were excavated. Individuals are color-coded by chronological period: Initial Jomon (8500–5000 BC) in orange, Early Jomon (5000–3520 BC) in purple, Middle Jomon (3520–2470 BC) in pink, Late Jomon (2470–1250 BC) in brown, and Final Jomon (1250–500 BC) in yellow.

The following figures are provided as separate files:

Figure S10. Non-symmetrical genetic affinities between Shomura individual and temporally and geographically diverse Jomon individuals.

Figure S11. Non-symmetrical genetic affinities Neshiko11 individual and temporally and geographically diverse Jomon individuals.

Figure S12. Non-symmetrical genetic affinities between the combined data of Neshiko13 and Neshiko16 individuals and temporally and geographically diverse Jomon individuals.
